# Supplementary material for: Comprehensive noise reduction in single-cell data with the RECODE platform
Source: Cell Rep Methods. 2025 Sep 17;5(10):101178. doi: 10.1016/j.crmeth.2025.101178 (PMC12570323; doi:10.1016/j.crmeth.2025.101178)
Supplement: Document S1. Figures S1–S6 [file mmc1.pdf]

Cell Reports Methods, Volume *5*

Supplemental information

Comprehensive noise reduction in single-cell data with the RECODE platform

Yusuke Imoto

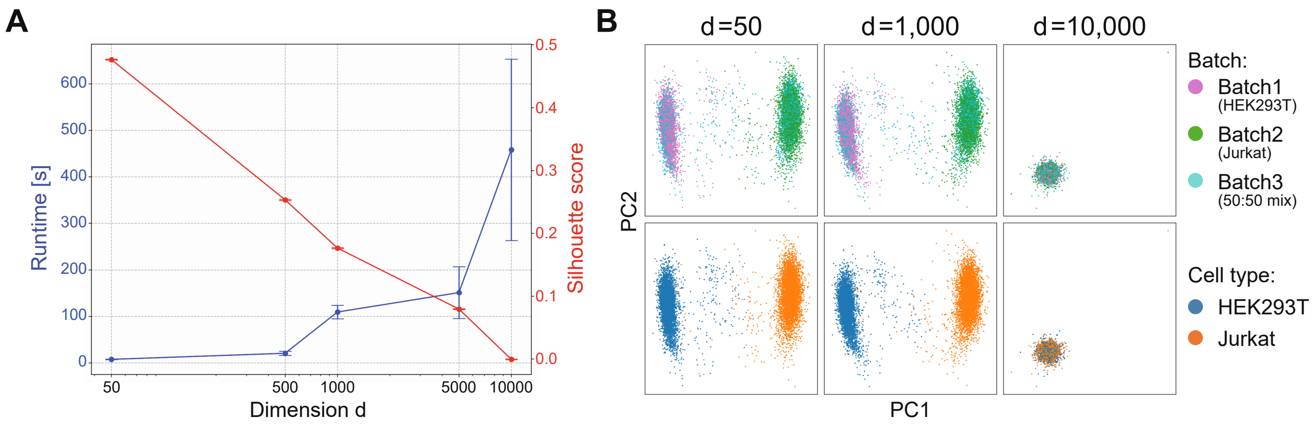

Figure S1: Impact of dimensionality on the performance of batch correction in single-cell data analysis, related to Figure 1. (A) A dual-axis plot demonstrating the performance of the batch correction method, Harmony, on scRNA-seq data from three batches containing HEK293T and Jurkat cell lines. The computational runtime, indicated by the blue line and plotted against the left vertical axis, shows an exponential increase with the dimensionality of the data. Conversely, the silhouette score for cell type, indicating the accuracy of batch correction, depicted by the red line and plotted against the right vertical axis, decreases as dimensionality increases, indicating that the batch correction method is also affected by the curse of dimensionality. The error bars represent standard deviations from 10 independent computations. (B) PCA projections illustrating the separation of cell types and batches at different data dimensionality. The plots reveal that higher dimensionality can degrade the clarity of cell type discrimination within batch-corrected data. These findings emphasize the necessity of dimensionality reduction as a preprocessing of batch correction, highlighting the difficulty in combining batch correction and technical noise reduction methods.

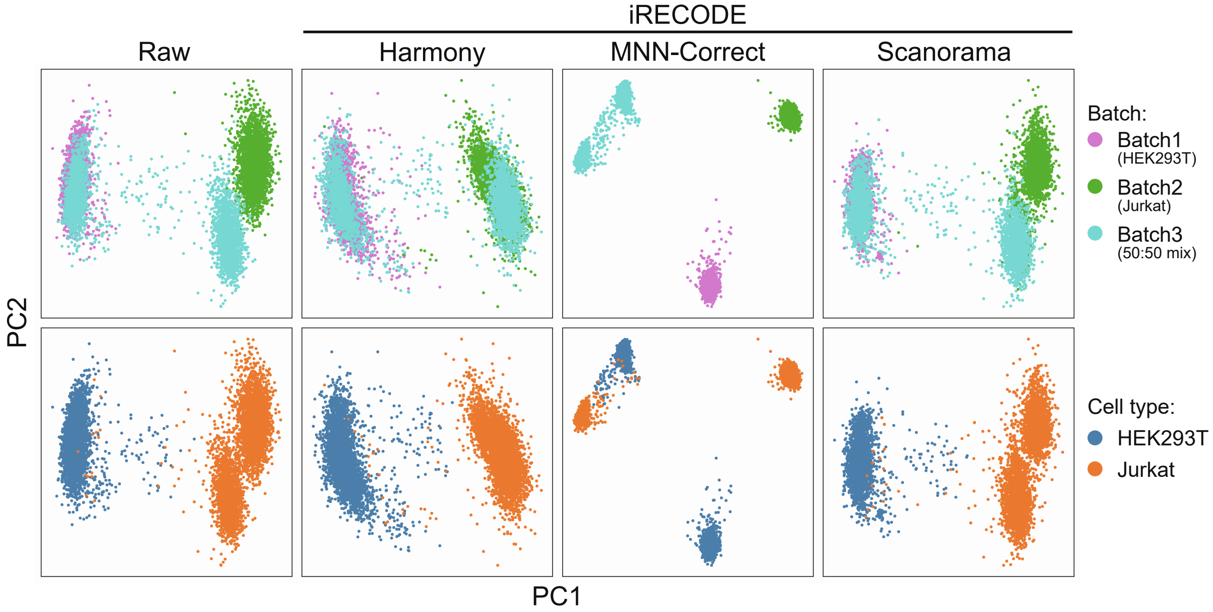

Figure S2: **Compatibility of batch correction methodologies in iRECODE, related to Figure 1.** This figure illustrates the performance of iRECODE incorporating three prominent batch correction algorithms, Harmony, MNN correct, and Scanorama, across datasets encompassing three batches and two cell lines (HEK293T and Jurkat). The first column represents the raw, uncorrected data, whereas the subsequent columns depict the data post-iRECODE using each method along the first two principal components (PC1 and PC2). iRECODE incorporating Harmony is notably effective in integrating the batches while maintaining cell type distinctions, as evidenced by the aligned clusters that segregate according to cell type rather than batch, underscoring its utility in harmonizing single-cell data while preserving biological variability.

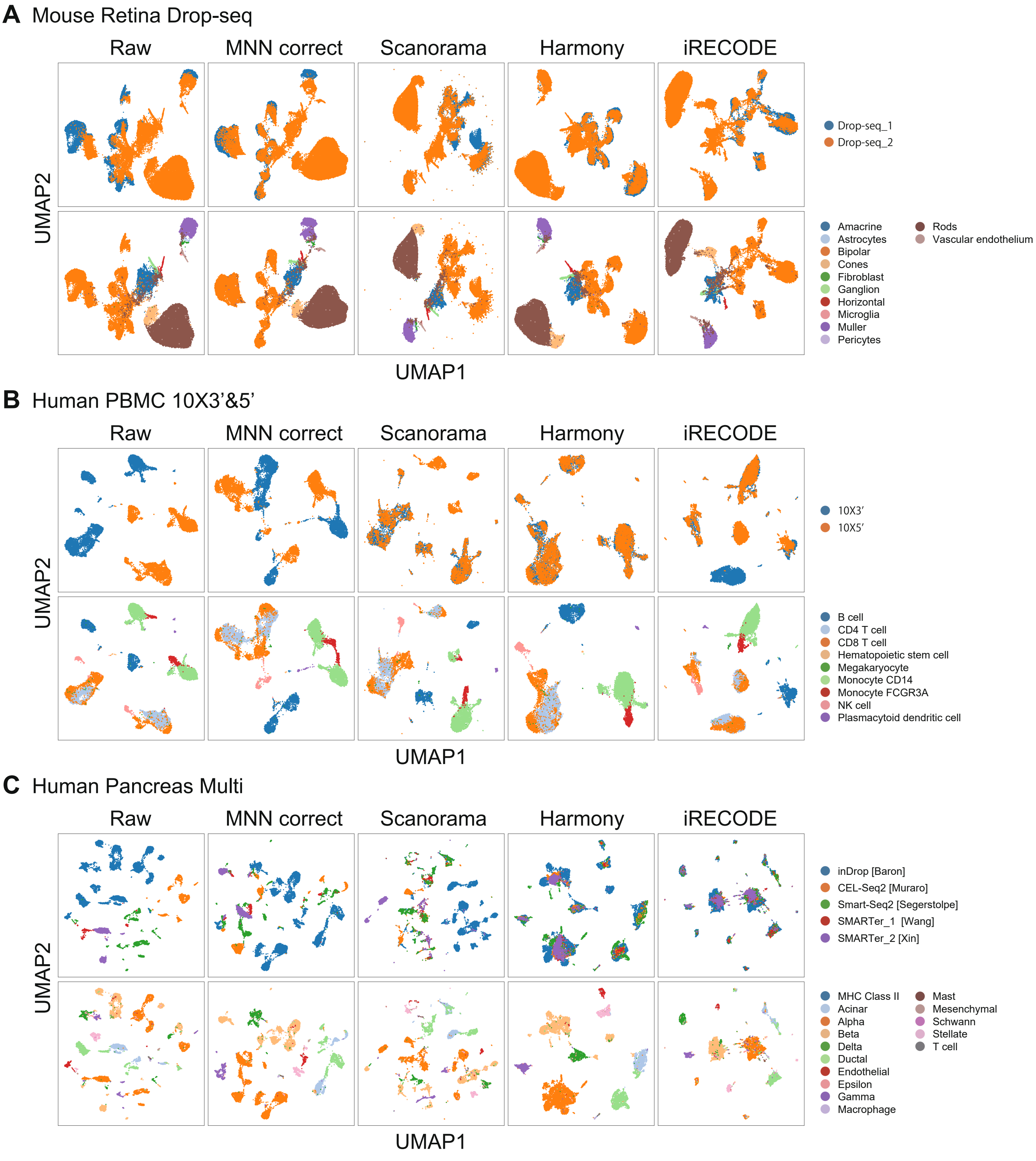

Figure S3: **Comparative visualization of scRNA-seq data integration using iRECODE across multiple datasets and platforms, related to Figure 2.** (A and B and C) UMAP projections demonstrating batch and cell-type distribution within mouse retina data obtained via Drop-seq (A) human peripheral blood mononuclear cells (PBMC) sequenced using 10X Genomics' 3' and 5' chemistry (B) and human pancreas cells sequenced using multiple single-cell technologies (C) with and without batch correction by various algorithms, including MNN correct, Scanorama, Harmony, and iRECODE.

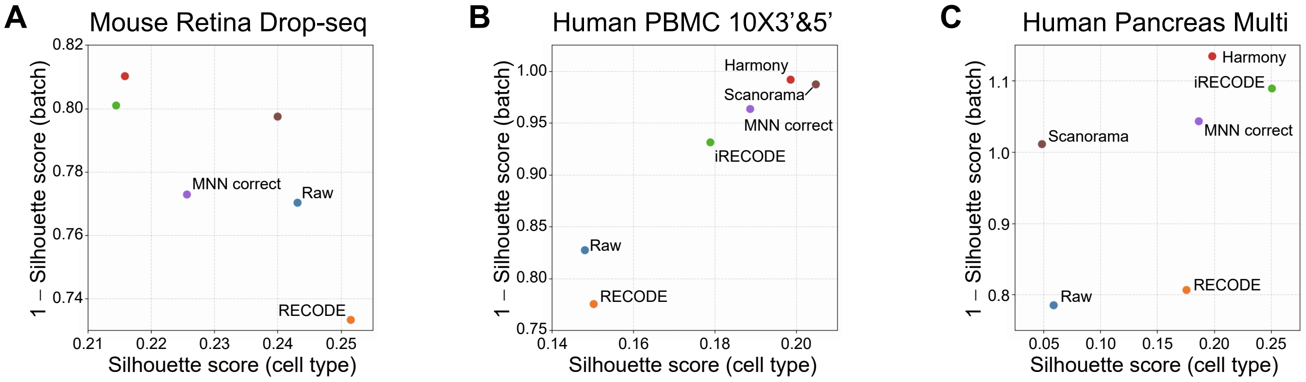

Figure S4: **Quantitative assessment of batch correction efficacy using silhouette scores across multiple datasets in Figure S3, related to Figure 2.** (A, B and C) Scatter plot illustrating the silhouette scores for cell type versus batch correction in the mouse retina Drop-seq dataset (A) the human PBMC 10X 3' and 5' dataset (B) and the human pancreas multi-platform dataset (C). Various batch correction methods, including MNN correct, Scanorama, Harmony, and iRECODE, are compared against the raw and original RECODE. Each point represents the score of a particular method, with the proximity to the top right indicating superior batch correction performance. While iRECODE consistently outperforms the original RECODE, its performance, like that of other batch correction methods, can vary depending on the dataset. This observation suggests that the effectiveness of iRECODE, as well as that of conventional batch correction approaches, depends on the specific characteristics and quality of the data, highlighting the importance of selecting appropriate methods based on the dataset context.

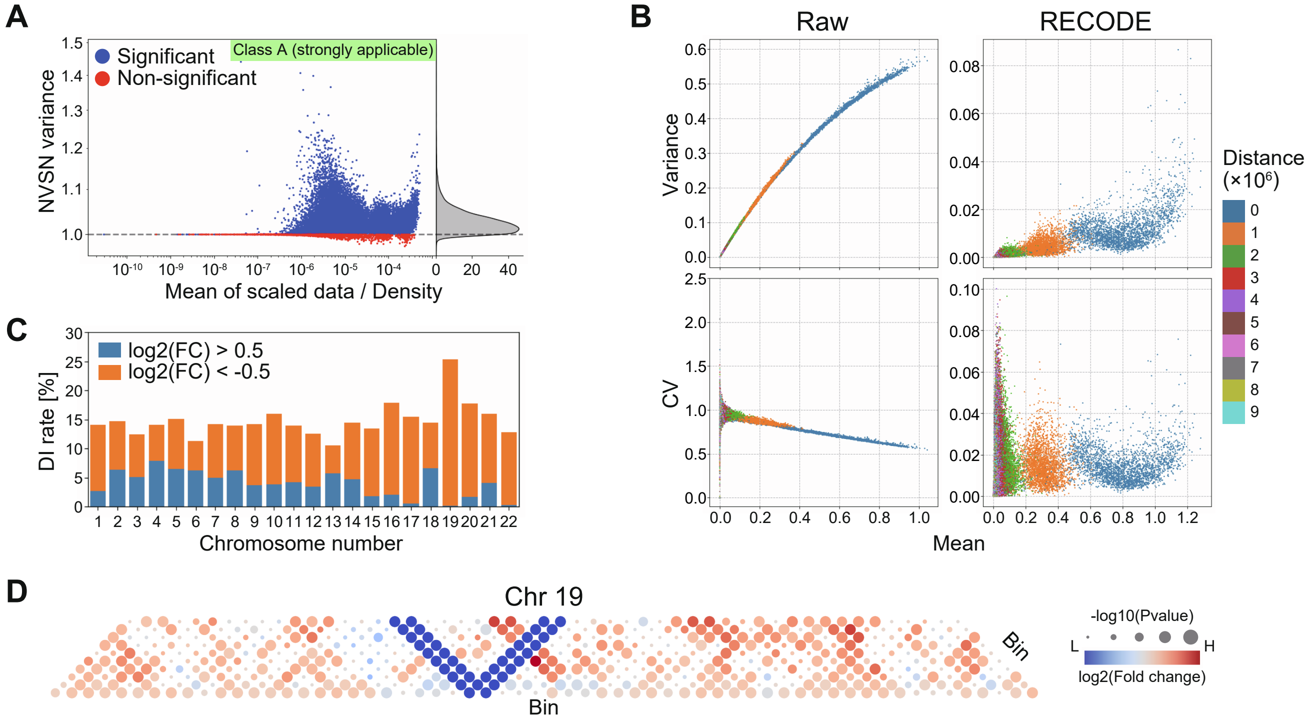

Figure S5: **scHi-C data analysis via RECODE, related to Figure 4.** (A) NVSN plot demonstrating RECODE's suitability for noise reduction of scHi-C data. (B) Comparative plots of variance and coefficient of variation (CV) against the mean for raw and RECODE-processed scHi-C data, with color gradation representing inter-bin distances. (C) Bar chart depicting the percentage of bins within differential interaction (DI) regions across all chromosomes, differentiated by the direction of log2 fold change (FC), providing insights into the chromosomal distribution of DIs defined in Figure 4F. (D) Spatial representation of DIs along chromosome 19, with circle size indicating the magnitude of $-\log(p\text{-value})$ and color denoting log2(FC), visualizing the regions of significant chromosomal interactions. These visualizations collectively quantify the impact of RECODE processing on the clarity and interpretability of scHi-C data, facilitating a deeper understanding of chromatin organization.

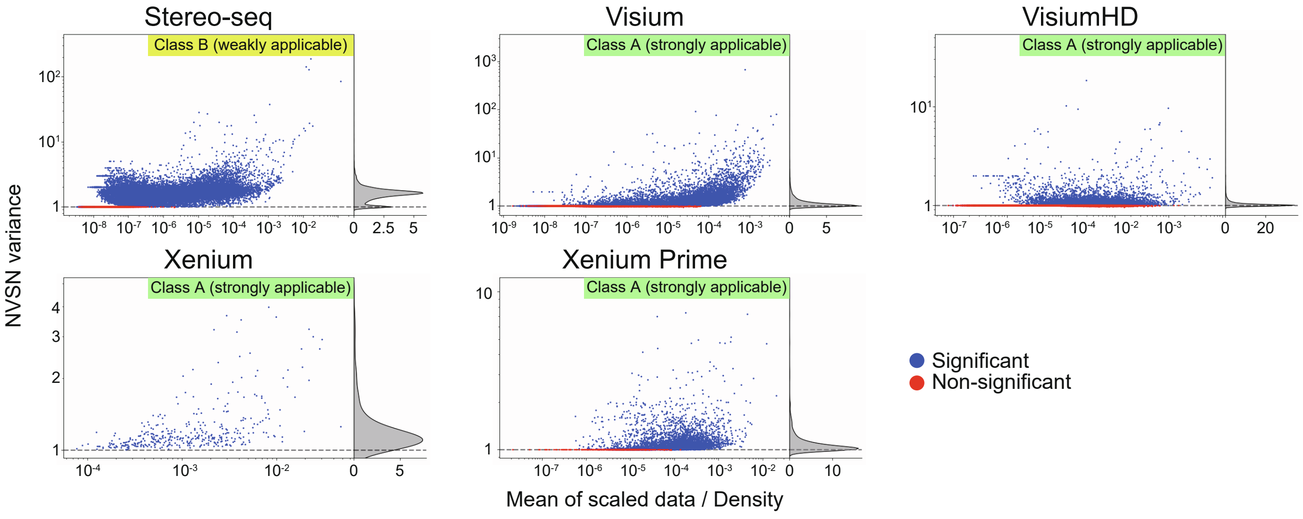

Figure S6: **Assessment of RECODE applicability to spatial transcriptomics datasets, related to Figure 5.** Spatial transcriptomics data from 10X Visium, 10X Visium HD, 10X Xenium, and Xenium Prime were classified as Class A (strongly applicable), whereas data from Stereo-seq were classified as Class B (weakly applicable).
