# Supplementary material for: Comprehensive noise reduction in single-cell data with the RECODE platform
Source: Cell Rep Methods. 2025 Sep 17;5(10):101178. doi: 10.1016/j.crmeth.2025.101178 (PMC12570323; doi:10.1016/j.crmeth.2025.101178)
Supplement: Document S2. Article plus supplemental information [file mmc2.pdf]

Cell Reports
Methods

Article

Comprehensive noise reduction in single-cell data with the RECODE platform

Graphical abstract

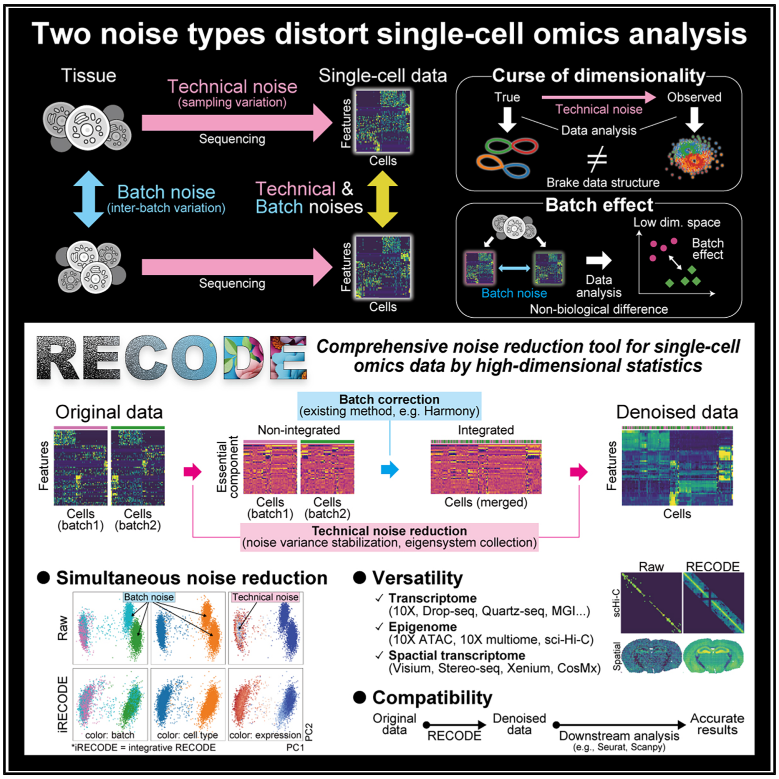

Authors

Yusuke Imoto

Correspondence

imoto.yusuke.4e@kyoto-u.ac.jp

In brief

Imoto presents an upgraded RECODE platform that simultaneously removes technical and batch noise while preserving full-dimensional single-cell data. Applicable to transcriptomic, epigenomic, and spatial datasets, RECODE improves accuracy, speed, and compatibility with existing workflows, enabling reliable detection of rare cell types and subtle biological variations.

Highlights

- RECODE simultaneously reduces technical and batch noise in single-cell data
- RECODE effectively denoises epigenomic (scHi-C) and spatial transcriptomics data
- Statistical innovations improve RECODE's accuracy and computational efficiency
- RECODE-denoised data integrate seamlessly with existing downstream analyses

Imoto, 2025, Cell Reports Methods 5, 101178
October 20, 2025 © 2025 The Author. Published by Elsevier Inc.
https://doi.org/10.1016/j.crmeth.2025.101178

CellPress

Cell Reports Methods

CellPress
OPEN ACCESS

Article

Comprehensive noise reduction in single-cell data with the RECODE platform

Yusuke Imoto[1,2,*]

[1]Institute for the Advanced Study of Human Biology (ASHBi), Kyoto University, Kyoto, Japan

[2]Lead contact

*Correspondence: imoto.yusuke.4e@kyoto-u.ac.jp

https://doi.org/10.1016/j.crmeth.2025.101178

MOTIVATION Single-cell omics technologies generate molecular profiles with unprecedented detail, but the data are often obscured by technical noise and batch effects. These artifacts mask subtle biological signals, hinder reproducibility, and limit the scope of downstream analyses. While our previous method, RECODE, successfully reduced technical noise, it could not address batch effects, and existing integration methods often compromise gene-level information through dimensionality reduction. To overcome these limitations, RECODE was upgraded to simultaneously reduce both technical and batch noise while preserving full-dimensional data, enabling more accurate and versatile single-cell analyses across diverse omics modalities.

SUMMARY

Single-cell sequencing enables genome- and epigenome-wide profiling of thousands of individual cells, offering unprecedented biological insights. However, technical noise and batch effects obscure high-resolution structures, hindering rare-cell-type detection and cross-dataset comparisons. To comprehensively address these challenges, this study upgrades RECODE, a high-dimensional statistics-based tool for technical noise reduction in single-cell RNA sequencing (RNA-seq), to include a function called iRECODE, which simultaneously reduces technical and batch noise. Further, RECODE's applicability is extended to diverse single-cell modalities, including single-cell high-throughput chromosome conformation capture (Hi-C) and spatial transcriptomics. Recent improvements in the algorithm have substantially enhanced both accuracy and computational efficiency. The RECODE platform thus provides a robust and versatile solution for noise mitigation, enabling more accurate downstream analyses across transcriptomic, epigenomic, and spatial domains.

INTRODUCTION

Single-cell sequencing technologies have driven a paradigm shift in genomics by enabling the resolution of genomic and epigenomic information at an unprecedented single-cell scale. The emergence of expansive single-cell databases, such as the Human Cell Atlas[1] and Tabula Sapiens,[2] underscores the urgent need for integrating single-cell data analysis across multiple genomic and epigenomic datasets.

However, the full potential of these datasets remains unrealized due to technical noise (dropout) and batch effects, which confound data interpretation.[3] Technical noise is a non-biological fluctuation caused by the non-uniformity of detection rates of molecules. It masks true cellular expression variability and complicates the identification of subtle biological signals. This effect has been demonstrated in several studies, where high dropout rates have been shown to obscure important biological phenomena, such as tumor-suppressor events in cancer[4] and cell-type-specific transcription factor activities.[5] Moreover, the high dimensionality of single-cell data introduces the curse of dimensionality, which obfuscates the true data structure under the effect of accumulated technical noise.[6] Batch effects further exacerbate analytical challenges by introducing non-biological variability across different datasets, stemming from minute differences in experimental conditions and sequencing platforms. These variations manifest as batch effects that distort comparative analyses and impede the consistency of biological insights across datasets.

Despite the numerous proposed techniques for technical noise reduction (imputation) and batch correction (integration),[7,8] the simultaneous reduction of both noise types remains a challenge. This difficulty arises primarily because conventional batch correction methods typically rely on dimensionality reduction, such as principal-component analysis (PCA), to reduce computational complexity. These methods often require pairwise distance calculations between cells across batches, where the computational burden increases with data dimensionality. Although PCA is used to reduce this dimensionality, it does not resolve the fundamental problem that high-dimensional noise degrades the reliability of such corrections. Indeed, it has been mathematically

This is an open access article under the CC BY license (http://creativecommons.org/licenses/by/4.0/).

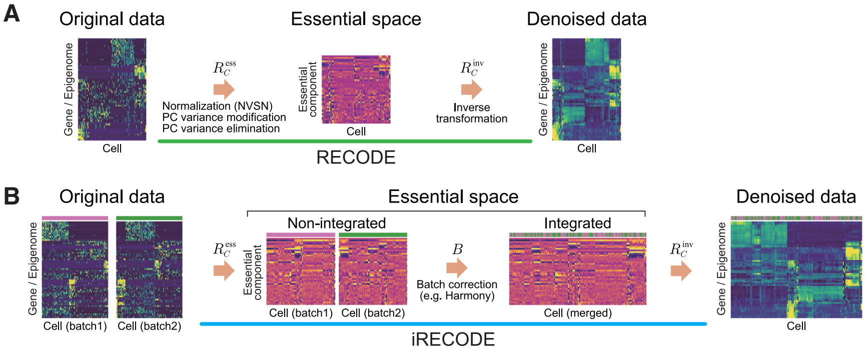

Figure 1. Schematic representation of the RECODE platform for comprehensive noise reduction in single-cell sequencing data

(A) The RECODE algorithm initiates the noise reduction process by mapping the original single-cell sequencing data into an essential space through noise variance-stabilizing normalization (NVSN). This series of processes, denoted as R_C^{ess}, attenuates technical noise via the principal-component (PC) variance modification and elimination. The subsequent stage involves an inverse transformation, represented by R_C^{inv}, which transposes the denoised data back to the original gene expression space.

(B) Enhancing the RECODE platform, iRECODE incorporates a batch correction step, *B*, within the essential space to address the batch noise. This addition enables us to align multiple batches released from the sparsity and the curse of dimensionality into a singular analytical platform, thus dramatically enhancing the accuracy of downstream data analyses.

demonstrated that dimensionality reduction techniques, including PCA, are insufficient to overcome the curse of dimensionality.[9] Consequently, simply combining technical noise reduction with batch-correction methodologies fails to effectively mitigate both types of noise in single-cell data analyses.

Previously, we developed the RECODE (resolution of the curse of dimensionality) algorithm, which models technical noise, arising from the entire data generation process from lysis through sequencing, as a general probability distribution, including the negative binomial distribution, and reduces it using an eigenvalue modification theory rooted in high-dimensional statistics.[10] Since then, RECODE has consistently outperformed other representative imputation methods regarding accuracy, speed, and practicability (parameter free), therefore representing a significant advance in the technical noise reduction of single-cell sequencing data. However, although RECODE was effective in resolving issues associated with technical noise, it neglected the effects of batch noise.

In this study, I establish a comprehensive noise reduction approach that further enhances the RECODE algorithm, making it capable of not only mitigating both technical and batch noise while preserving data dimensions but also processing various types of single-cell sequencing data, including epigenomics and spatial transcriptomics datasets. Moreover, by improving its accuracy and computational speed, RECODE is now a versatile platform capable of resolving large-scale integrative single-cell analyses that span multiomics layers and cell types, thereby enhancing our ability to address more complex biological phenomena.

RESULTS

Enhancing RECODE for dual noise reduction of single-cell sequencing data

As single-cell sequencing gives rise to two common problems, namely technical noise, which includes dropout events, and batch effects, to simultaneously address these issues, I developed iRECODE (integrative RECODE). This method synergizes the high-dimensional statistical approach of RECODE[10] with the established batch correction approach. The original RECODE maps gene expression data to an essential space using noise variance-stabilizing normalization (NVSN) and singular value decomposition and then applies principal-component variance modification and elimination (Figure 1A; STAR Methods). As the accuracy and computational efficiency of most batch-correction methods decline as the dimensionality increases (Figure S1), iRECODE was designed to integrate batch correction within this essential space, thereby minimizing the decrease in accuracy and the increase in computational cost by bypassing high-dimensional calculations (Figure 1B; STAR Methods). This enabled us to implement a simultaneous reduction in technical and batch noise with low computational costs. In addition, iRECODE allows the selection of any batch-correction method within its platform.

I used single-cell RNA sequencing (scRNA-seq) data comprising three datasets and two cell lines[8,11] to compare the compatibility of three prominent batch-correction algorithms, Harmony,[12] MNN-correct,[13] and Scanorama,[14] with iRECODE. The results indicated that Harmony performed the best for batch correction (Figure S2), and I therefore used Harmony as the batch correction in the iRECODE algorithm hereafter. The application of iRECODE successfully mitigated batch effects, as evidenced by improved cell-type mixing across batches and elevated integration scores based on the local inverse Simpson's index (iLISI) while preserving distinct cell-type identities as indicated by stable cLISI values, both of which were comparable to those achieved with Harmony, a state-of-the-art batch-correction method (Figures 2A and 2B). In addition, iRECODE reduced sparsity in the gene expression matrix and substantially lowered dropout rates, resulting in clearer and more continuous expression patterns across cells (Figures 2A and 2C). Thus, iRECODE presents a robust approach for the simultaneous reduction of technical and batch noise in single-cell data analyses.

Advancing toward authentic single-cell transcriptome analysis with iRECODE

Next, I quantitatively evaluate the performance of iRECODE in comparison to established methods for reducing technical and batch noise using the dataset of the previous section. iRECODE refined the gene expression distributions and, accordingly, also addressed dropout and sparsity, mirroring the efficacy of the original RECODE method (Figure 3A). iRECODE notably

CellPress
OPEN ACCESS

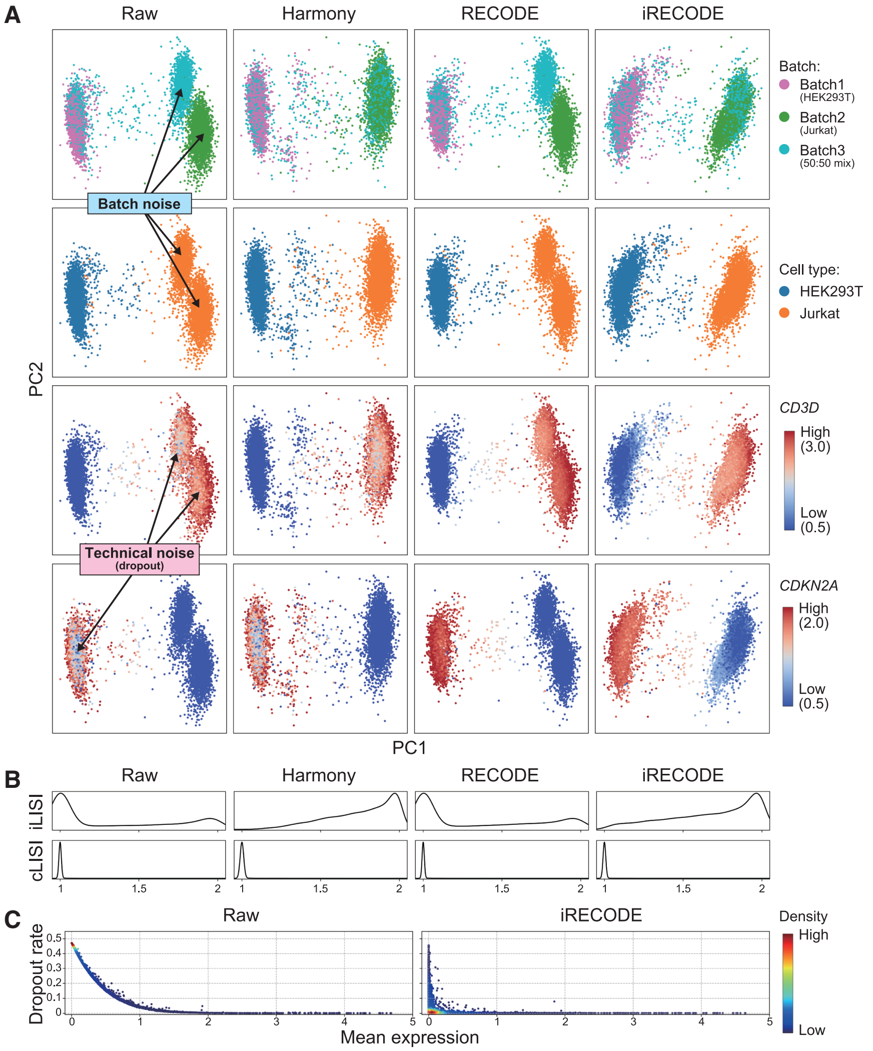

Figure 2. Efficacy of iRECODE in mitigating technical and batch noise in scRNA-seq data

(A) Principal-component analysis (PCA) results of scRNA-seq data comparing three batches and two cell types in various conditions: unprocessed (raw) and processed with Harmony, with RECODE, and with iRECODE. The third and fourth rows show gene expression levels of well-established marker genes: *CD3D* for Jurkat cells[11] and *CDKN2A* for HEK293T cells,[15] respectively.

(B) Assessment of integration quality using distributions of local inverse Simpson's index for both batch integration (iLISI) and cell-type conservation (cLISI).

(C) Dropout rates against mean expression values before and after iRECODE. The raw data show distinct batch effects and technical noise, leading to the non-biological segregation and misannotation of clusters by batch and gene expression sparsity. RECODE and Harmony each independently ameliorate a specific aspect of noise, but iRECODE synergistically harmonizes the datasets, resulting in consistent integration across cell types (B) and a marked reduction in dropout events (C), illustrating the comprehensive capabilities of iRECODE in enhancing data fidelity for downstream analysis.

modulates the variance among non-housekeeping genes while consistently diminishing the variance among housekeeping genes, indicating a successful reduction in technical noise (Figure 3B).

Improvements in batch noise correction were substantial, with the relative errors in the mean expression values significantly decreasing from 11.1% to 14.3% to a mere 2.4%–2.5% (Figure 3A; STAR Methods). On a genomic scale, iRECODE notably enhanced the relative error metrics by over 20% and 10% from those of raw data and traditional RECODE-processed data, respectively (Figure 3C). Moreover, performance comparisons with established batch-correction methods using the silhouette score show that iRECODE is as effective as Harmony, MNN-correct, and Scanorama, demonstrating its accuracy (Figure 3D).

The precision of iRECODE could also be applied to datasets produced by various scRNA-seq technologies, including Drop-seq, Smart-seq, and multiple 10× Genomics protocols (Figures S3 and S4). Despite the greater computational load due to the preservation of data dimensions, iRECODE was approximately ten times more efficient than the combination of technical noise reduction and batch-correction methods (Figure 3E). Together, these results establish iRECODE as a viable and comprehensive approach for noise reduction in single-cell analysis.

Application of RECODE to single-cell epigenomics and spatial transcriptomics

The capabilities of RECODE extend beyond scRNA-seq, offering a promising solution for the inherent technical noise present in other data types derived from similar random sampling mechanisms, such as scATAC-seq and single-cell high-throughput chromosome conformation capture (scHi-C) for epigenomics and spatial transcriptomics. These methodologies rely on random molecular sampling using sequencing, akin to scRNA-seq. This section demonstrates the versatility of RECODE in processing and refining single-cell epigenomics and spatial transcriptomics datasets, underscoring its broad applicability and potential to enhance data accuracy across various single-cell sequencing platforms.

Refining single-cell Hi-C data analysis with RECODE

scHi-C data, presenting a matrix of contact frequencies within chromosomes, offer insights into understanding cell-specific epigenomic architecture distinct from transcriptomic data. However, the sparsity inherent in scHi-C data requires robust noise reduction strategies to enable meaningful cell annotation and significant interaction detection. As the noise generated between scHi-C and scRNA-seq datasets is similar, this suggests

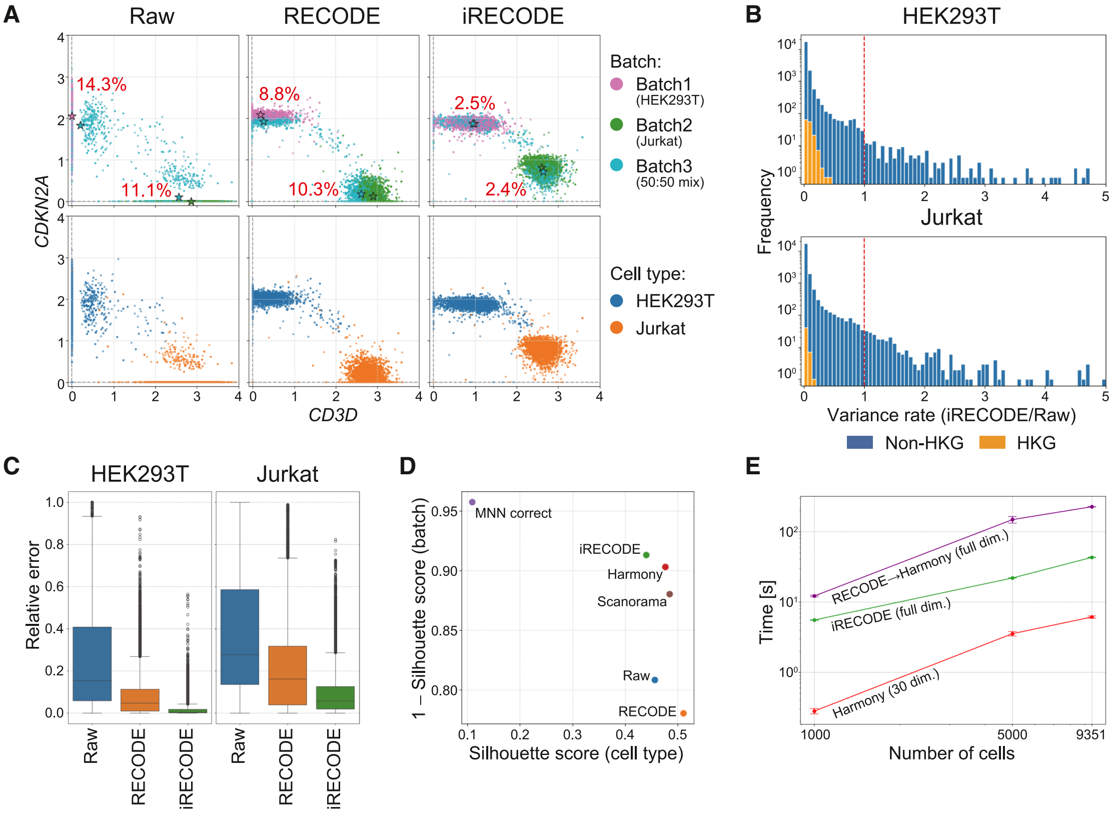

Figure 3. Quantitative assessment and comparative analysis of iRECODE for scRNA-seq data

(A) Scatterplots presenting gene expression of marker genes across different batches and cell types in raw and RECODE- and iRECODE-processed data. Stars indicate the centroid of gene expression for each group, with percentages reflecting the batch-effect-reflected relative errors between centroids per cell type.

(B) Histograms displaying the variance ratio of gene expression between iRECODE-integrated and raw data, with blue representing non-housekeeping genes (non-HKGs) and orange denoting housekeeping genes (HKGs).

(C) Boxplots demonstrating the distribution of the relative errors, calculated based on differences in average gene expression across batches, reflecting the batch effect for HEK293T and Jurkat cell lines across processing states: raw, RECODE, and iRECODE.

(D) Silhouette scores quantifying cell-type homogeneity and batch-effect mitigation, with the scores indicating improved data integration by iRECODE compared to the raw and prominent batch-correction method processed data.

(E) The computational runtime for iRECODE, Harmony, and the combination of original RECODE and Harmony is analyzed across varying cell numbers, illustrating the scalability and efficiency of iRECODE. Input data dimensionality was set as 21,474 genes. The error bars indicate the standard deviation based on 10 independent calculations.

that RECODE may be effective in reducing noise associated with scHi-C data. To test whether RECODE could discern differential interactions (DIs) that define cell-specific interactions, it was applied to the matrix data constructed by vectorizing the upper triangle of scHi-C contact maps using sci-Hi-C datasets from five human cell lines at 1 Mbp resolution.[16] The NVSN distribution, which is an indicator for the applicability of RECODE, revealed that scHi-C data affected by technical noise can be effectively processed using RECODE (Figure S5A). Indeed, RECODE considerably mitigated data sparsity, aligning the scHi-C-derived topologically associating domains (TADs) with their bulk Hi-C counterparts (Figures 4A and 4B). Raw scHi-C data, constrained by variances and coefficients of variation influenced by bin distances and mean values, obscured the heterogeneity of interactions among cells (Figure S5B). However, RECODE-processed data revealed significant interactions that more accurately reflected this heterogeneity, thereby overcoming the limitations of the raw data. Subsequent PCA and uniform manifold approximation and projection (UMAP) analyses of the processed data confirmed distinct cell-type segregation (Figures 4C and 4D).

To quantitatively evaluate the effectiveness of RECODE in capturing cell-type-specific structures in scHi-C data, I compared silhouette scores across several methods, including PCA, latent Dirichlet allocation (LDA), and RECODE (Figure 4E). Despite retaining the full dimensionality of the original data, RECODE outperformed PCA and achieved a silhouette score comparable to LDA, which has been reported as a method for extracting cell-type-specific chromatin compartment patterns from scHi-C data.[16] Furthermore, when RECODE was combined with LDA, the silhouette score increased even further, indicating that RECODE preprocessing enhances the effectiveness of downstream dimensionality reduction and clustering techniques.

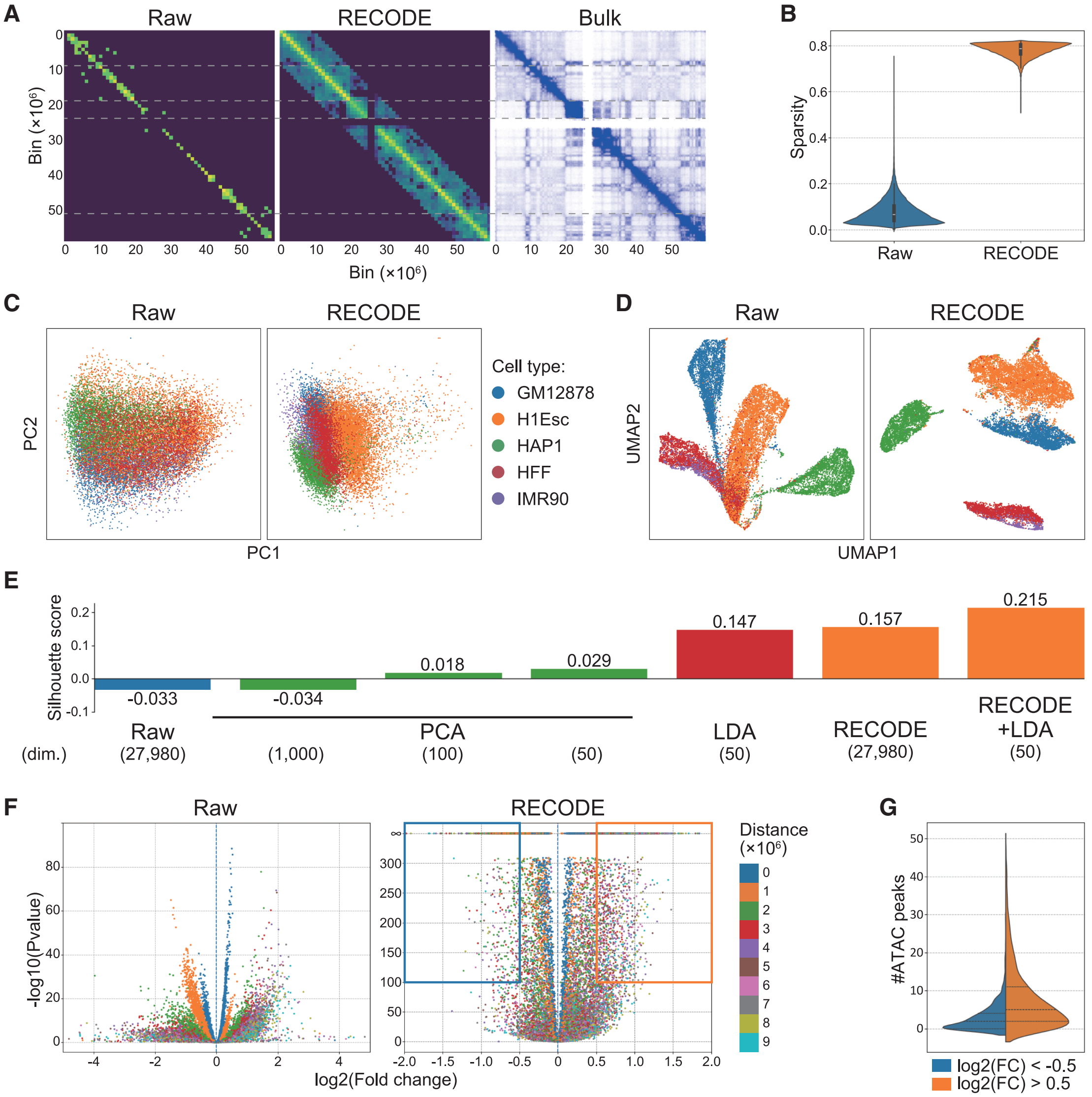

Figure 4. Evaluation of RECODE's impact on scHi-C data interpretation

(A) Comparison of contact maps at 1 Mbp resolution for raw, RECODE-processed scHi-C data, and bulk Hi-C in GM12878 cells, delineating chromosomal interactions at chromosome 19.

(B) Violin plots illustrating the non-zero interaction rate (sparsity) for raw versus RECODE-processed scHi-C data, highlighting RECODE's ability to alleviate data sparsity.

(C and D) Dimensionality reduction visualizations, with PCA and UMAP, for raw and RECODE-processed scHi-C data, colored by cell type, indicating enhanced data segregation post-RECODE processing.

(E) Average silhouette scores of cell types for raw, PCA-dimensionally reduced, LDA-dimensionally reduced, RECODE-processed, and RECODE- and LDA-processed scHi-C data, illustrating improved clustering quality with RECODE. Bracketed numbers indicate the dimensionality of each dataset.

(F) Volcano plots contrasting differential interactions (DIs) within GM12878 cells against other cell types, with color coding based on inter-bin distances. These plots emphasize the distribution of significant interactions, with the right section (log2[fold change] [log2(FC)] > 0) spotlighting GM12878-specific DIs. Interaction specificity for GM12878 cells is marked by the highlighted orange and blue box regions.

(G) Distribution of the count of ATAC peaks for GM12878 cells, categorized based on bins within boxes in (F), demonstrating the disparity in chromatin accessibility associated with DIs.

In the downstream DI analysis, while raw scHi-C data struggled to detect significant DIs owing to the drop in detection rate corresponding to the distance between bins, RECODE precisely identified cell-type-specific DIs that overlapped with ATAC-seq-derived open chromatin regions (Figures 4F and 4G). Notably, RECODE showed that chromosome 19 had significant cell-type-specific interactions (Figures S5C and S5D). Thus, RECODE effectively denoises single-cell epigenome sequencing data under the same principles used for single-cell transcriptome data, thereby

Table 1. Classification of the applicability of RECODE across diverse single-cell sequencing platforms

	Strongly applicable	Weakly applicable	Inapplicable
Transcriptome			
10× (RNA)	✓	–	–
Drop-seq	✓	–	–
Quartz-seq	✓	–	–
inDrop	✓	–	–
CEL-Seq2	✓	–	–
DNBSEQ (MGISEQ)	✓	–	–
Smart-seq 2	–	✓	–
Smart-seq 3	–	✓	–
Fluidigm C1	–	✓	–
SC3-seq	–	✓	–
TAS-seq	–	✓	–
SMARTer	–	–	✓
Epigenome			
10× (ATAC)	✓	–	–
Sci-Hi-C	✓	–	–
Multiome			
10× (RNA+ATAC)	✓	–	–
CITE-seq	✓	–	–
Spatial transcriptomics			
10× Visium (HD)	✓	–	–
Stereo-seq	–	✓	–
10× Xenium	✓	–	–
CosMx	–	✓	–

revealing intricate cell-specific chromosomal and epigenomic landscapes.

Refining spatial transcriptomics analysis with RECODE

Spatial transcriptomics analysis significantly enhances our understanding of cellular heterogeneity and interactions within native tissue environments and provides critical insights into the complex relationships of biological systems. Similar to other sequencing technologies, it faces the challenge of technical noise, which can mask biological signals and obstruct key discoveries. Because the gene expression part of the spatial transcriptomics data is generated through mechanisms similar to those of other sequencing technologies, the application of RECODE, a method for denoising the technical noise generated in the sequencing process, can effectively resolve this issue.

I applied RECODE to gene expression data from spatial transcriptomics datasets generated using the Stereo-seq, 10× Visium, and 10× Xenium platforms. Based on the NVSN distributions, these datasets exhibited technical noise patterns well modeled by RECODE (Figure S6; Table 1). Although Stereo-seq offers near-single-cell resolution, raw data sparsity remains a notable limitation (Figures 5A and 5B). The application of RECODE improved the distributions, revealing clearer spatial gene expression patterns. To quantitatively assess this improvement, I calculated Welch's t values by comparing gene expression between target and non-target cell regions for each marker gene. Across all seven tested genes, RECODE substantially increased t values compared to raw data, indicating stronger spatial specificity and more reliable identification of marker gene expression domains (Figure 5B). To further evaluate the versatility of RECODE, I applied it to spatial transcriptomics datasets from multiple platforms, including 10× Visium, Visium HD, Xenium, and Xenium Prime (Figure 5C). Despite differences in resolution, species, tissue types, and target genes, RECODE consistently enhanced spatial signal clarity and reduced data sparsity. These results underscore the broad applicability of RECODE across diverse spatial transcriptomics technologies.

Enhancing accuracy and reducing computational costs of RECODE

Recent advances in high-dimensional statistical theory have substantially bolstered the noise reduction capabilities of RECODE. By incorporating Yata-Aoshima's theoretical eigenvalue modification,[18,19] the original RECODE addresses the curse of dimensionality.[10] They also introduced an approach to modify eigenvectors using sparse PCA theory,[20] which I integrated into the updated version of RECODE. This eigenvector modification, occurring simultaneously with the eigenvalue modification within the essential space of RECODE, further mitigates technical noise that remains in the essential space of RECODE (STAR Methods). This update results in the preservation of variation in marker genes and a reduction in housekeeping genes, indicating an enhancement in the detection of key differentially expressed genes (Figures 6A and 6B).

To further improve the practicality of RECODE, I also incorporated a downsampling learning (DL) approach. This allows RECODE to learn denoising functions from a randomly selected subset of single-cell data, thereby preserving the need for full dataset utilization (STAR Methods). By applying this fast RECODE algorithm, computational efficiency was greatly improved without sacrificing accuracy. With a modest 20% of the data employed in the learning phase, the DL algorithm markedly accelerated the operational speed of RECODE, outpacing other previous computations (Figures 6C and 6D). Moreover, this algorithm showed near-first-order scalability concerning both the cellular and feature dimensions (Figures 6C and 6D), thereby ensuring efficiency in the face of emerging big datasets.

DISCUSSION

In this study, I developed an integrative preprocessing methodology for single-cell sequencing data based on the advancement of our noise reduction technique, RECODE. Grounded on the principles of high-dimensional statistics, RECODE addresses the pervasive issue of technical noise commonly associated with dropout events in single-cell sequencing data. The evolved methodology, which I have called iRECODE, expands this capability to encompass batch noise attenuation. This is achieved by integrating batch correction directly within the algorithm's essential low-dimensional space, giving rise to a method that

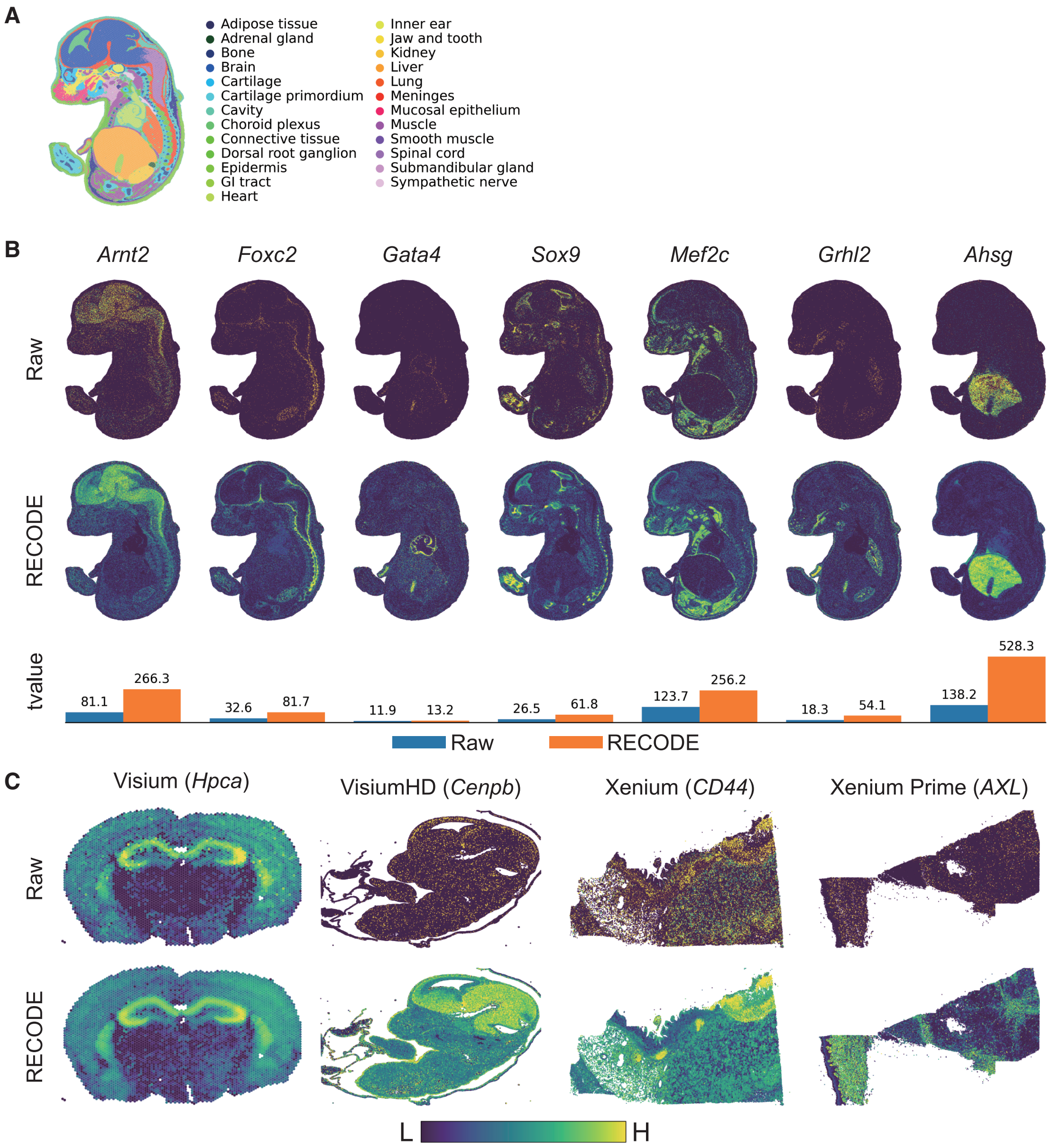

Figure 5. Illustration of RECODE's denoising efficacy on spatial transcriptomics data

(A) Annotated cell-type spatial distribution within a mouse embryo at embryonic day 16.5 defined in Chen et al.[17]

(B) Side-by-side visualization of spatial gene expression patterns and Welch's *t* values for selected cell-type-specific markers in the mouse embryo, contrasting raw data with RECODE-processed data.

(C) Gene expression maps before and after RECODE processing across multiple cell types and spatial transcriptomics platforms: mouse brain (10× Visium), mouse embryo (10× Visium HD), human pancreatic ductal adenocarcinoma (10× Xenium), and human skin (10× Xenium Prime). Gene names are shown in parentheses.

not only improves the precision of noise reduction but also substantially decreases computational demand. Additionally, Harmony was found to be the most compatible for batch correction with iRECODE, possibly due to it not assuming a gene expression space and therefore allowing for batch correction in RECODE's essential space.

I also showed that the utility of RECODE goes beyond transcriptomics, encompassing both single-cell epigenomics and spatial transcriptomics, by distinguishing cell types using chromosomal architecture and resolving spatial patterns of low-expression genes, respectively. Furthermore, enhancements in the accuracy and processing speed of RECODE have further enabled it to handle the influx of large-scale datasets, ensuring faster and more precise noise reduction. These advances significantly broaden the applications for RECODE, establishing it as a fundamental tool that will become the

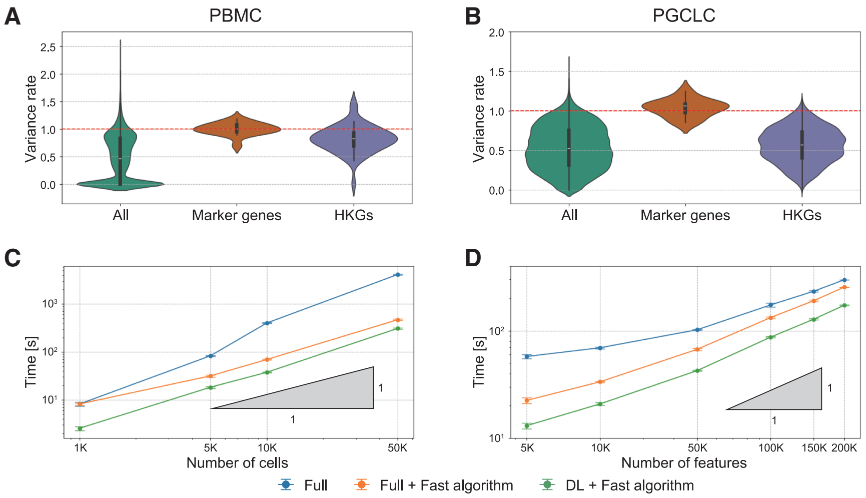

Figure 6. Assessment of RECODE's enhanced algorithmic accuracy and computational performance

(A and B) Violin plots of variance ratios for peripheral blood mononuclear cell (PBMC) and primordial germ cell-like cell (PGCLC) datasets, respectively, comparing gene expression variability across all genes, marker genes, and housekeeping genes (HKGs) between the original and the improved RECODE algorithms with eigenvector modification. The dashed red line indicates equal variance between the two versions.

(C and D) Computational time required by the RECODE algorithm relative to the number of cells and features analyzed. Performance metrics compare the naive algorithm using all cells (full), an established fast algorithm (STAR Methods), and a downsampling learning (DL) strategy combined with the fast algorithm. Standard deviations are denoted by error bars, while the triangles represent linear scalability in processing time. These visualizations quantify the advancements in RECODE's algorithmic accuracy for biological marker gene detection and its computational efficiency, pivotal for large-scale single-cell data analysis.

foundation for single-cell data analysis across extensive datasets.

Looking back at the theory of RECODE, it models the data generation of single-cell sequencing as random sampling, removing noise while preserving the true gene expression distribution, thereby ensuring no loss of genuine biological signals. This approach avoids the cyclicity issue commonly observed in traditional imputation methods, in which improvements in certain aspects inadvertently lead to detrimental effects on others.[3] Additionally, RECODE is applicable to a wide range of sequencing platforms because of the noise modeling inherent in the sequencing process, without limiting specific molecules, cell types, or data generation platforms (Table 1). Furthermore, as RECODE retains the original data structure, including the matrix size and expression scale, the data processed by RECODE can be seamlessly integrated with conventional normalization and downstream analyses.[21] Therefore, RECODE will likely emerge as an essential preprocessing step in the analysis of all single-cell data in the future.

In sum, RECODE is a comprehensive and versatile platform for noise reduction in single-cell sequencing, with the potential to go beyond its current transcriptomic applications in epigenomics and spatial transcriptomics, revolutionizing our understanding of complex biological systems.

Limitations of the study

In scenarios such as early cell differentiation or cancer cells with relatively few mutations, where genetic information is overwhelmingly similar, traditional clustering may lead to errors even when non-biological noise is completely removed. Therefore, in post-noise reduction, the selection of biologically variable genes for classification becomes pivotal, potentially requiring supplementary methodologies such as ScType.[22]

Furthermore, distinguishing batch noise from slight biological variations remains a critical challenge—for example, subtle transcriptional changes associated with aging or stress can be easily confounded with batch effects. However, the flexibility in choosing batch-correction methods in iRECODE suggests that as research in this area progresses, so will the capabilities of iRECODE.

Despite its current capabilities, further refinement of RECODE applications will be an important future goal. Some datasets classified as weakly applicable indicate the presence of nonrandom sampling noise (Table 1). Furthermore, good applicability has also been shown with non-sequence-based data, such as fluorescence *in situ* hybridization (FISH)-based single-cell spatial transcriptomics (Xenium and CosMx). One potential solution is the mathematical enhancement of the noise variance model, which can further improve the utility of RECODE. Additionally, the application of RECODE to diverse genomic and epigenomic datasets is expected to reveal biological insights that were previously concealed by noise.

RESOURCE AVAILABILITY

Lead contact

Requests for further information should be directed to and will be fulfilled by the lead contact, Yusuke Imoto (imoto.yusuke.4e@kyoto-u.ac.jp).

Materials availability

This manuscript contains no unique reagents or resources.

Data and code availability

- All datasets used in this study are publicly available from previously published resources and repositories, including GEO (GEO: GSE140021), NCBI SRA (SRA: SRP073767), ENCODE3 (ENCODE: ENCSR095QNB), the MOSTA database, 10× Genomics datasets (Visium, Visium HD, Xenium, and Xenium Prime), and other sources as indicated in the key resources table.
- The RECODE source code is available at GitHub (https://github.com/yusuke-imoto-lab/RECODE) and has been archived at Zenodo (https://doi.org/10.5281/zenodo.16916446).
- Any additional information required to reanalyze the data reported in this paper is available from the lead contact upon request.

ACKNOWLEDGMENTS

I extend my sincere appreciation to Drs. Cantas Alev, Yasuaki Hiraoka, Masahiro Nagano, Tomonori Nakamura, Mitinori Saitou, Taro Tsujimura, and Kazuyoshi Yata for their invaluable discussions and insights, which have greatly contributed to the advancement of this work. I would also like to thank Dr. Spyros Goulas for critical comments and constructive suggestions on my manuscript. This research was supported by the World Premier International Research Center Initiative (WPI), MEXT, Japan; the JST PRESTO program (grant number JPMJPR2021); the JST FOREST program (grant number JPMJFR222X); and the JST CREST program (grant number JPMJCR24Q1), which provided funding and an inspirational research environment.

AUTHOR CONTRIBUTIONS

Conceptualization, Y.I.; methodology, Y.I.; investigation, Y.I.; writing – original draft, Y.I.; writing – review & editing, Y.I.; funding acquisition, Y.I.; supervision, Y.I.

DECLARATION OF INTERESTS

Y.I. is an inventor on a patent application relating to RECODE filed by Kyoto University.

DECLARATION OF GENERATIVE AI AND AI-ASSISTED TECHNOLOGIES IN THE WRITING PROCESS

During the preparation of this work, the author used ChatGPT for the purpose of language refinement and improvement of clarity in the draft manuscript. After using this tool, the author carefully reviewed, revised, and edited the content as necessary and takes full responsibility for the content of the publication.

STAR★METHODS

Detailed methods are provided in the online version of this paper and include the following:

- KEY RESOURCES TABLE
- METHOD DETAILS
  - RECODE
  - iRECODE
  - Eigenvector modification for accuracy improvement
  - Downsampling learning (DL) for acceleration
- QUANTIFICATION AND STATISTICAL ANALYSIS
  - Integration analysis
  - scHi-C data analysis
  - Spatial transcriptomics analysis
  - Verifications of accuracy and computational costs

SUPPLEMENTAL INFORMATION

Supplemental information can be found online at https://doi.org/10.1016/j.crmeth.2025.101178.

Received: August 22, 2024
Revised: May 23, 2025
Accepted: August 22, 2025
Published: September 17, 2025

REFERENCES

1. Regev, A., Teichmann, S.A., Lander, E.S., Amit, I., Benoist, C., Birney, E., Bodenmiller, B., Campbell, P., Carninci, P., Clatworthy, M., et al. (2017). The human cell atlas. eLife *6*, e27041. https://doi.org/10.7554/eLife.27041.
2. Tabula Sapiens Consortium*; Jones, R.C., Karkanias, J., Krasnow, M.A., Pisco, A.O., Quake, S.R., Salzman, J., Yosef, N., Bulthaup, B., Brown, P., et al. (2022). The tabula sapiens: A multiple-organ, single-cell transcriptomic atlas of humans. Science *376*, eabl4896. https://doi.org/10.1126/science.abl4896.
3. Lähnemann, D., Köster, J., Szczurek, E., Mccarthy, D.J., Hicks, S.C., Robinson, M.D., Vallejos, C.A., Campbell, K.R., Beerenwinkel, N., Mahfouz, A., et al. (2020). Eleven grand challenges in single-cell data science. Genome Biol. *21*, 31. https://doi.org/10.1186/s13059-020-1926-6.
4. Teschendorff, A.E., and Wang, N. (2020). Improved detection of tumor suppressor events in single-cell rna-seq data. NPJ Genom. Med. *5*, 43. https://doi.org/10.1038/s41525-020-00151-y.
5. Maity, A.K., Hu, X., Zhu, T., and Teschendorff, A.E. (2022). Inference of age-associated transcription factor regulatory activity changes in single cells. Nat. Aging *2*, 548–561. https://doi.org/10.1038/s43587-022-00233-9.
6. Hall, P., Marron, J.S., and Neeman, A. (2005). Geometric representation of high dimension, low sample size data. J. Roy. Stat. Soc. B Stat. Methodol. *67*, 427–444. https://doi.org/10.1111/j.1467-9868.2005.00510.x.
7. Hou, W., Ji, Z., Ji, H., and Hicks, S.C. (2020). A systematic evaluation of single-cell rna-sequencing imputation methods. Genome Biol. *21*, 218. https://doi.org/10.1186/s13059-020-02132-x.
8. Tran, H.T.N., Ang, K.S., Chevrier, M., Zhang, X., Lee, N.Y.S., Goh, M., and Chen, J. (2020). A benchmark of batch-effect correction methods for single-cell rna sequencing data. Genome Biol. *21*, 12. https://doi.org/10.1186/s13059-019-1850-9.
9. Yata, K., and Aoshima, M. (2009). Pca consistency for non-gaussian data in high dimension, low sample size context. Commun. Stat. Theor. Methods *38*, 2634–2652. https://doi.org/10.1080/03610910902936083.
10. Imoto, Y., Nakamura, T., Escolar, E.G., Yoshiwaki, M., Kojima, Y., Yabuta, Y., Katou, Y., Yamamoto, T., Hiraoka, Y., and Saitou, M. (2022). Resolution of the curse of dimensionality in single-cell RNA sequencing data analysis. Life Sci. Alliance *5*, e202201591. https://doi.org/10.26508/lsa.202201591.
11. Zheng, G.X.Y., Terry, J.M., Belgrader, P., Ryvkin, P., Bent, Z.W., Wilson, R., Ziraldo, S.B., Wheeler, T.D., McDermott, G.P., Zhu, J., et al. (2017). Massively parallel digital transcriptional profiling of single cells. Nat. Commun. *8*, 14049. https://doi.org/10.1038/ncomms14049.
12. Korsunsky, I., Millard, N., Fan, J., Slowikowski, K., Zhang, F., Wei, K., Baglaenko, Y., Brenner, M., Loh, P.R., and Raychaudhuri, S. (2019). Fast, sensitive and accurate integration of single-cell data with harmony. Nat. Methods *16*, 1289–1296. https://doi.org/10.1038/s41592-019-0619-0.
13. Haghverdi, L., Lun, A.T.L., Morgan, M.D., and Marioni, J.C. (2018). Batch effects in single-cell rna-sequencing data are corrected by matching mutual nearest neighbors. Nat. Biotechnol. *36*, 421–427. https://doi.org/10.1038/nbt.4091.
14. Hie, B., Bryson, B., and Berger, B. (2019). Efficient integration of heterogeneous single-cell transcriptomes using scanorama. Nat. Biotechnol. *37*, 685–691. https://doi.org/10.1038/s41587-019-0113-3.
15. Liang, J., Fan, J., Wang, M., Niu, Z., Zhang, Z., Yuan, L., Tai, Y., Chen, Z., Song, S., Wang, X., et al. (2018). Cdkn2a inhibits formation of homotypic cell-in-cell structures. Oncogenesis *7*, 50. https://doi.org/10.1038/s41389-018-0056-4.
16. Kim, H.J., Yardımcı, G.G., Bonora, G., Ramani, V., Liu, J., Qiu, R., Lee, C., Hesson, J., Ware, C.B., Shendure, J., et al. (2020). Capturing cell type-specific chromatin compartment patterns by applying topic modeling to single-cell Hi-C data. PLoS Comput. Biol. *16*, e1008173. https://doi.org/10.1371/journal.pcbi.1008173.
17. Chen, A., Liao, S., Cheng, M., Ma, K., Wu, L., Lai, Y., Qiu, X., Yang, J., Xu, J., Hao, S., et al. (2022). Spatiotemporal transcriptomic atlas of mouse organogenesis using dna nanoball-patterned arrays. Cell *185*, 1777–1792.e21. https://doi.org/10.1016/j.cell.2022.04.003.
18. Yata, K., and Aoshima, M. (2010). Effective pca for high-dimension, low-sample-size data with singular value decomposition of cross data matrix.

J. Multivariate Anal. *101*, 2060–2077. https://doi.org/10.1016/j.jmva.2010.04.006.

19. Yata, K., and Aoshima, M. (2012). Effective pca for high-dimension, low-sample-size data with noise reduction via geometric representations. J. Multivariate Anal. *105*, 193–215. https://doi.org/10.1016/j.jmva.2011.09.002.
20. Yata, K., and Aoshima, M. (2025). Automatic sparse pca for high-dimensional data. Stat. Sin. *35*, 1069–1090. https://doi.org/10.5705/ss.202022.0319.
21. Imoto, Y. (2025). Accurate highly variable gene selection using recode in scrna-seq data analysis. Preprint at bioRxiv. https://doi.org/10.1101/2025.06.23.661026.
22. Ianevski, A., Giri, A.K., and Aittokallio, T. (2022). Fully-automated and ultra-fast cell-type identification using specific marker combinations from single-cell transcriptomic data. Nat. Commun. *13*, 1246. https://doi.org/10.1038/s41467-022-28803-w.
23. Hounkpe, B.W., Chenou, F., de Lima, F., and De Paula, E.V. (2021). Hrt atlas v1.0 database: redefining human and mouse housekeeping genes and candidate reference transcripts by mining massive rna-seq datasets. Nucleic Acids Res. *49*, D947–D955. https://doi.org/10.1093/nar/gkaa609.
24. Robinson, J.T., Turner, D., Durand, N.C., Thorvaldsdóttir, H., Mesirov, J.P., and Aiden, E.L. (2018). Juicebox.js provides a cloud-based visualization system for Hi-C data. Cell Syst. *6*, 256–258.e1. https://doi.org/10.1016/j.cels.2018.01.001.
25. Chen, D., Sun, N., Hou, L., Kim, R., Faith, J., Aslanyan, M., Tao, Y., Zheng, Y., Fu, J., Liu, W., et al. (2019). Human primordial germ cells are specified from lineage-primed progenitors. Cell Rep. *29*, 4568–4582.e5. https://doi.org/10.1016/j.celrep.2019.11.083.
26. Kojima, Y., Sasaki, K., Yokobayashi, S., Sakai, Y., Nakamura, T., Yabuta, Y., Nakaki, F., Nagaoka, S., Woltjen, K., Hotta, A., et al. (2017). Evolutionarily distinctive transcriptional and signaling programs drive human germ cell lineage specification from pluripotent stem cells. Cell Stem Cell *21*, 517–532.e5. https://doi.org/10.1016/j.stem.2017.09.005.
27. Wolf, F.A., Angerer, P., and Theis, F.J. (2018). Scanpy: large-scale single-cell gene expression data analysis. Genome Biol. *19*, 15. https://doi.org/10.1186/s13059-017-1382-0.

STAR★METHODS

KEY RESOURCES TABLE

REAGENT or RESOURCE	SOURCE	IDENTIFIER
Deposited data		
Integration benchmark datasets (datasets 4, 5, 6, 7)	Tran et al.[8]	https://github.com/JinmiaoChenLab/Batch-effect-removal-benchmarking
Sci-Hi-C data	Noble Lab	https://noble.gs.washington.edu/proj/schic-topic-model/
Mouse embryo Stereo-seq (MOSTA database)	Chen et al.[17]	https://db.cngb.org/stomics/mosta/
Mouse brain, Visium	10x Genomics	Visium CytAssist Gene Expression Libraries of Post-Xenium Mouse Brain (FF)
Mouse embryo, Visium HD	10x Genomics	Visium HD Spatial Gene Expression Library, Mouse Embryo (FFPE)
Human pancreatic ductal adenocarcinoma, Xenium	10x Genomics	FFPE Human Pancreatic Ductal Adenocarcinoma Data with Human Immuno-Oncology Profiling Panel
Human skin, Xenium Prime	10x Genomics	FFPE Human Skin Primary Dermal Melanoma with 5K Human Pan Tissue and Pathways Panel
Human GM12878 and mouse A20, scATAC-seq	10x Genomics	5k 1:1 Mixture of Fresh Frozen Human (GM12878) and Mouse (A20) Cells
68k PBMCs, scRNA-seq	NCBI SRA	SRP073767
Human PGCLC induction system, scRNA-seq	GEO	GSE140021
GM12878 cells, bulk ATAC-seq	ENCODE3	ENCSR095QNB
Software and algorithms		
RECODE (Python and R implementations)	This paper; GitHub/Zenodo	https://github.com/yusuke-imoto-lab/RECODE; https://doi.org/10.5281/zenodo.16916446

METHOD DETAILS

RECODE

Here, I provide an overview of the RECODE algorithm.[10] I first introduced noise variance-stabilizing normalization (NVSN), which is the first step in the RECODE algorithm. Let $\mathbb{N}$, $\mathbb{N}_0$, and $\mathbb{R}$ be sets of natural numbers, non-negative integers, and real values, respectively. Let $C = (c_{ij}) \in \mathbb{N}_0^{d\times n}$ be the count data matrix, such as the raw single-cell genome and epigenome sequencing data, which are the inputs of RECODE, where d and n are the dimensions (number of features) and number of cells, respectively. I define $t_j = \sum_i c_{ij}$ as the total count of cells j and remove cells with $t_j = 0$ from the input data in advance. Then, we define NVSN $F_C : \mathbb{R}^{d\times n} \to \mathbb{R}^{d\times n}$ as

$$[F_C(X)]_{ij} := \begin{cases} \dfrac{x_{ij}}{t_j\sqrt{n^{-1}\sum_k c_{ik} t_k^{-2}}}, & \sum_k c_{ik} > 0, \\ 0, & \sum_k c_{ik} = 0, \end{cases}$$

where $X = (x_{ij}) \in \mathbb{R}^{d\times n}$. I introduced $F_C(C)$ the NVSN matrix.

Next, the transformation of the RECODE algorithm is introduced. For matrix $X = (x_{ij}) \in \mathbb{R}^{d\times n}$, let $S_X := (X - XP)(X - XP)^{\mathsf{T}}/(n-1) \in \mathbb{R}^{d\times d}$ be the covariance matrix of X, where P is an $n \times n$ matrix whose elements are all $1/n$ (XP shows the row-wise mean vector). Further, let $U_X = (u_{X,1}, \ldots, u_{X,d}) \in \mathbb{R}^{d\times d}$ and $\Lambda_X = \mathrm{diag}(\lambda_{X,1}, \ldots, \lambda_{X,d}) \in \mathbb{R}^{d\times d}$ $(\lambda_{X,1} \geq \ldots \geq \lambda_{X,d})$ be the components of the singular value decomposition of S_X, i.e., $S_X = U_X \Lambda_X U_X^{\mathsf{T}}$. Here, $(\lambda_{X,i}, u_{X,i})$ denotes the pair of i th eigenvalue and eigenvector of the covariance matrix S_X. Furthermore, $u_{X,i}$ and $\lambda_{X,i}$ correspond to the i th axis and variance of PCA, respectively. Yata and Aoshima proposed a modification of the PC variance as

$$\tilde{\lambda}_{X,i} := \begin{cases} \lambda_{X,i} - \dfrac{1}{\min\{n-1,d\} - i+1} \displaystyle\sum_{k=i+1}^{\min\{n-1,d\}} \lambda_{X,k}, & i = 1, \ldots, \ell, \\ 0, & \text{otherwise} \end{cases} \quad \text{(Equation 1)}$$

and proved an improvement in its convergence in a high-dimensional statistical setting.[19] Here, $\ell \leq \min\{n-1, d\}$ is a parameter called the essential dimension of the matrix X. I propose the optimal value ℓ^* of the essential dimension for the normalized matrix $F_C(C)$ as:

$$\ell^* := \min\left\{k; \sum_{i=k+1}^{d} \lambda_{F_C(C),i} \leq (d-k)\right\}.$$

Then, I define the RECODE transformation $R_C : \mathbb{R}^{d\times n} \rightarrow \mathbb{R}^{d\times n}$ for the count data matrix C as:

$$R_C(X) := F_C^{-1}\left[U_{F_C(C),\ell^*}\tilde{\Lambda}^{1/2}_{F_C(C),\ell^*}\Lambda^{-1/2}_{F_C(C),\ell^*}U^{\mathrm{T}}_{F_C(C),\ell^*}[F_C(X) - F_C(C)P] + F_C(C)P\right]. \quad \text{(Equation 2)}$$

Here, $U_{X,k} = (u_{X,1},\dots,u_{X,k}) \in \mathbb{R}^{d\times k}$, $\tilde{\Lambda}^{1/2}_{X,k} = \mathrm{diag}(\tilde{\lambda}^{1/2}_{X,1},\dots,\tilde{\lambda}^{1/2}_{X,k}) \in \mathbb{R}^{k\times k}$, and $\Lambda^{-1/2}_{X,k} = \mathrm{diag}(\lambda^{-1/2}_{X,1},\dots,\lambda^{-1/2}_{X,k}) \in \mathbb{R}^{k\times k}$. $R_C(C)$ is the RECODE output matrix.

Applicability: RECODE determines the suitability of noise reduction efforts through a robust analysis of gene variance within the NVSN matrix, stratifying datasets into three distinct categories: strongly applicable, weakly applicable, and inapplicable.[10] This classification is based on a mathematical theorem concerning the distribution of variances in the NVSN matrix: A dataset is classified as strongly applicable when the variance distribution is tightly concentrated around 1, weakly applicable when the variance distribution is broadly dispersed above 1, and inapplicable when the distribution deviates substantially from these patterns. Strong applicability indicates congruence with the RECODE noise model, which is conducive to substantial technical noise mitigation. Weak applicability, while still within the RECODE platform, suggests the presence of additional non-modeled noise. However, inapplicability indicates a fundamental discrepancy with the model, in which the noise deviates from that accounted for by random sampling. This systematic categorization informs the tailored application of RECODE across diverse single-cell sequencing datasets with detailed Table 1.

Fast algorithm: The fast RECODE algorithm was implemented using the algebraic characteristics between the matrices and eigenvalues.[10] Calculating the average of the subsequent eigenvalues in the traditional PC variance modification requires a full PCA computation. We derived an equivalent algorithm that utilized only a subset of eigenvalues by exploiting the relationship between the trace of the matrix and the eigenvalue. This innovation permits the use of truncated PCA instead of a full PCA, thereby substantially enhancing the computational efficiency of RECODE. Moreover, the combination of a fast algorithm and downsampling learning introduced in the subsequent section represents a significant leap forward in the speed of the algorithm, streamlining the noise reduction process for large-scale single-cell datasets.

iRECODE

iRECODE unifies multiple datasets in the essential space of RECODE. Using transformations $R_C^{\mathrm{ess}} : \mathbb{R}^{d\times n} \rightarrow \mathbb{R}^{\ell^*\times n}$ and $R_C^{\mathrm{inv}} : \mathbb{R}^{\ell^*\times n} \rightarrow \mathbb{R}^{d\times n}$, defined as

$$R_C^{\mathrm{ess}}(X) := \tilde{\Lambda}^{1/2}_{F_C(C),\ell^*}\Lambda^{-1/2}_{F_C(C),\ell^*}U^{\mathrm{T}}_{F_C(C),\ell^*}[F_C(X) - F_C(C)P],$$
$$R_C^{\mathrm{inv}}(Y) := F^{-1}\left[U_{F_C(C),\ell^*}Y + F_C(C)P\right],$$

RECODE transformation R_C is represented as $R_C = R_C^{\mathrm{inv}} \circ R_C^{\mathrm{ess}}$. I call $R_C^{\mathrm{ess}}(C)$ in $\mathbb{R}^{\ell^*\times n}$ the essential matrix of RECODE. Batch correction in the essential space reduces the computational cost because the essential dimension ℓ^* is usually $10 \sim 1{,}000$. In summary, using batch correction $B : \mathbb{R}^{\ell^*\times n} \rightarrow \mathbb{R}^{\ell^*\times n}$, iRECODE transformation iR_C is formulated as

$$\begin{aligned} iR_C(X) \; &:= F_C^{-1}\left[U_{F_C(C),\ell^*}B\left[\tilde{\Lambda}^{1/2}_{F_C(C),\ell^*}\Lambda^{-1/2}_{F_C(C),\ell^*}U^{\mathrm{T}}_{F_C(C),\ell^*}[F_C(X) - F_C(C)P]\right] + F_C(C)P\right] \\ &= R_C^{\mathrm{inv}} \circ B \circ R_C^{\mathrm{ess}}(X). \end{aligned}$$

$iR_C(C)$ is the output matrix of the iRECODE.

Eigenvector modification for accuracy improvement

RECODE employs an eigenvalue modification of the covariance matrix as a pivotal step in the PC variance adjustment, a method based on the study by Yata and Aoshima.[18,19] A recent methodological advancement by Yata and Aoshima introduced an eigenvector modification technique[20] that leverages the principles of sparse PCA. This technique refines the eigenvectors by automatically optimizing the parameters through an eigenvalue modification framework. I integrated this cutting-edge approach to increase the accuracy of the RECODE algorithm.

I formulated an eigenvector modification and combined it with the RECODE algorithm. Recall that $\lambda_{X,i}$ and $u_{X,i}$ are i th eigenvalue and eigenvector of covariance matrix $X \in \mathbb{R}^{d\times n}$, respectively. Furthermore, $\tilde{\lambda}_{X,i}$ is the modified eigenvalue defined in Equation 1. For each eigenvector $u_{X,i} = (u_{X,i1},\dots,u_{X,id})$, I define a symmetric group $\sigma : \{1,\dots,d\} \rightarrow \{1,\dots,d\}$ such that $|u_{X,i\sigma(1)}| \geq \dots \geq |u_{X,i\sigma(d)}|$. I set integer k_i such that

$$\sum_{j=1}^{k_i}\left[u_{X,i\sigma(j)}\right]^2 < \frac{\tilde{\lambda}_{X,i}}{\lambda_{X,i}}, \quad \sum_{j=1}^{k_i+1}\left[u_{X,i\sigma(j)}\right]^2 \geq \frac{\tilde{\lambda}_{X,i}}{\lambda_{X,i}}.$$

I then define the modified eigenvector $\tilde{u}_{X,i} = (\tilde{u}_{X,i1}, \ldots, \tilde{u}_{X,id})$ as

$$\tilde{u}_{X,ij} := \begin{cases} \dfrac{u_{X,ij}}{\sum_{\ell=1}^{k_i} \left[u_{X,i\sigma(\ell)}\right]^2}, & \sigma^{-1}(j) \leq k_i, \\ 0, & \sigma^{-1}(j) > k_i. \end{cases}$$

This transformation eliminated the components of the eigenvector that were close to zero based on the rate of modification of the eigenvalues. Using modified eigenvector matrix $\tilde{U}_{X,k} = (\tilde{u}_{X,1}, \ldots, \tilde{u}_{X,k}) \in \mathbb{R}^{d \times k}$, I define accuracy-improved RECODE transformation $\tilde{R}_C : \mathbb{R}^{d \times n} \rightarrow \mathbb{R}^{d \times n}$ as

$$\tilde{R}_C(X) := F_C^{-1}\left[\tilde{U}_{F_C(C),\ell^*} \tilde{\Lambda}^{1/2}_{F_C(C),\ell^*} \Lambda^{-1/2}_{F_C(C),\ell^*} \tilde{U}^{\mathrm{T}}_{F_C(C),\ell^*} [F_C(X) - F_C(C)P] + F_C(C)P\right].$$

In addition, I introduced an accuracy-improved iRECODE iR_C by replacing $U_{F_C(C),\ell^*}$ with $\tilde{U}_{F_C(C),\ell^*}$. Accuracy improvement was executed using the version = 2 option on the GitHub code.

Downsampling learning (DL) for acceleration

RECODE operates as an unsupervised learning algorithm that learns a transformation from the input count matrix C and applies it to the input data (refer to Equation 2). The RECODE algorithm incurs most of the computational costs during the learning process. To enhance efficiency, I implemented a downsampling strategy to expedite learning.

Consider n^* (where $n^* < n$) as the sample size after post-downsampling, and $C^* \in \mathbb{R}^{d \times n^*}$ as the corresponding downsampled count data. The downsampling learning algorithm for RECODE, denoted as R_{C^*}, learns transformations using C^* and subsequently applies these transformations to the original input data C, producing denoised data $R_{C^*}(C)$. Because the RECODE transformation relies on summary statistics such as the mean and variance of gene expression, it is robust as long as the number of cells remains sufficiently large after downsampling. Our GitHub repository for RECODE defaults to a 20% downsampling rate for datasets exceeding 20,000 samples.

QUANTIFICATION AND STATISTICAL ANALYSIS

Integration analysis

In our integration analysis, I used benchmark datasets 4–7 from Tran et al.[8] These included dataset 4: human pancreas analyzed via multiple protocols (Figures S2C and S3C), dataset 5: human pancreas using 3′ and 5′ 10X Chromium protocols (Figures S2B and S3B), dataset 6: cell line comparison (HEK293T and Jurkat) via Drop-seq (Figures 2 and 3), and dataset 7: mouse retina by Drop-seq (Figures S2A and S3A). iRECODE was applied to raw scRNA-seq data.

The dropout rate (Figure 2C) was quantified as the percentage of zero counts per gene. The relative error RE(i) for the i th gene between batches b1 and b2 (shown in Figures 3A and 3C) is defined as

$$\mathrm{RE}(i) := \frac{\left|\overline{x}_i^{\mathrm{b1}} - \overline{x}_i^{\mathrm{b2}}\right|}{\max\left\{\overline{x}_i^{\mathrm{b1}}, \overline{x}_i^{\mathrm{b2}}\right\}},$$

where $\overline{x}_i^{\mathrm{bj}}$ is the mean expression value of the i th gene in the j th batch. The relative errors were assessed for each cell type. Housekeeping genes (analyzed in Figure 3B) were identified using the HRT Atlas v1.0.[23] To evaluate computational efficiency (Figure 3E), the runtime was measured ten times for randomly selected cells ($n = 1,000, 5,000, 9,531$ (full)). The quantitative integration scores, including LISI and silhouette scores, are as follows:

LISI: The local inverse Simpson's index (LISI) quantitatively assesses the efficacy of batch correction by measuring the local diversity around each cell.[12] It is predicated on the variance in annotations among a cell's nearest neighbors. Integration LISI (iLISI) utilizes batch labels with distributions approaching the number of batches per cell type, indicating thorough mixing. Conversely, cell-type LISI (cLISI) is based on cell-type annotations, where distributions near one suggest the successful segregation of cell types.

Silhouette score: The silhouette score was used to calculate the goodness of the clustering technique. Its value ranges from -1 to 1, where a high value indicates that the object is well-matched to its own cluster and poorly matched to neighboring clusters. If most objects have high values, then the clustering configuration is appropriate. If many points have low or negative values, the clustering configuration may contain too many or too few clusters. The silhouette score is particularly useful when the ground truth of the data is unknown, and it provides a succinct graphical representation of how well each object lies within its cluster.

scHi-C data analysis

I utilized single-cell combinatorial-indexed Hi-C (sci-Hi-C) data at 1M-bp resolution from five human cell lines: GM12878, H1Esc, HFF, IMR90, and HAP1.[16] The observed contacts were limited to those within 10M-bp inter-bin distances. A matrix was constructed from the cell's scHi-C contact map by flattening the upper triangular portion. Matrix data were transformed using the RECODE algorithm.

Following RECODE processing, both raw and processed data were subjected to log normalization, setting the stage for downstream analyses. In addition, a bulk Hi-C contact map, as shown in Figure 4A, was generated using Juicebox.[24] In Figure 4F, I used bulk ATAC-seq data (ENCSR095QNB) of GM12878 cells downloaded from ENCODE3 data portal.

Spatial transcriptomics analysis

I employed Stereo-seq data from mouse embryos at E16.5[17] (E16.5_E1S1.MOSTA.h5ad) and demonstration data from 10X Visium for mouse brain and human colon cancer. RECODE was applied to the gene expression count matrices excluding the spatial coordinates. Similar to the approach used for scRNA-seq data, standard log-normalization was performed, followed by mapping of the processed gene expression values onto spatial coordinates. Welch's t-values in Figure 5B are defined by

$$\frac{\text{mean}(X_t) - \text{mean}(X_o)}{\sqrt{\text{var}(X_t)/n_t + \text{var}(X_o)/n_o}}$$

where X_t and X_o denote the expression values for the target cell type and all other cell types, respectively. The values n_t and n_o represent the number of samples for each group. The Welch's t-value indicates the statistical significance of the difference in means and is used to assess marker gene specificity. In Figure 5B, the target cell types were defined as follows: brain and spinal cord for *Arnt2*, meninges for *Foxc2*, heart and gastrointestinal tract for *Gata4*, choroid plexus for *Sox9*, muscle for *Mef2c*, lung and gastrointestinal tract for *Grhl2*, and liver for *Ahsg*. To ensure consistent and comparable visualization, the color scales used in Figures 5B and 5C were standardized across conditions. In Figure 5B, the minimum and maximum values of the color bar were set to the 0th and 99th percentiles of the gene expression values for each gene, while in Figure 5C, they were set to the 0th and 95th percentiles. The applicability of RECODE for Stereo-seq and 10X Visium data was determined to be weak and strong, respectively (Figure S6).

Verifications of accuracy and computational costs

For accuracy verification, I employed scRNA-seq data of peripheral blood mononuclear cells (PBMC) derived from 10X Genomics demonstration datasets for Figure 6A and human primordial germ cell-like cell (hPGCLC) induction system data on day 2[25] for Figure 6B. Marker genes for PBMCs included *CD14*, *CD79A*, *CD8A*, *CD8B*, *CST3*, *FCER1A*, *FCGR3A*, *GNLY*, *IL7R*, *LGALS3*, *LYZ*, *MS4A1*, *MS4A7*, *NKG7*, *S100A8*, *KLRB1*, and for hPGCLC system *GATA2*, *GATA3*, *GATA4*, *HAND1*, *MIXL1*, *NANOG*, *NANOS3*, *POU5F1*, *PRDM1*, *SOX17*, *TFAP2A*, *TFAP2C*, selected based on previous research.[25–27] Housekeeping genes were identified using HRT Atlas v1.0.[23]

For computational efficiency verification, I employed fresh scRNA-seq data of 68k PBMCs[11] for Figure 6C and scATAC-seq data of a 5k mix of human GM12878 and mouse A20 cells for Figure 6D generated using the 10X Chromium platform. Runtime measurements were conducted ten times for randomly selected cells or ATAC regions.

Cell Reports Methods, Volume *5*

Supplemental information

Comprehensive noise reduction in single-cell data with the RECODE platform

Yusuke Imoto

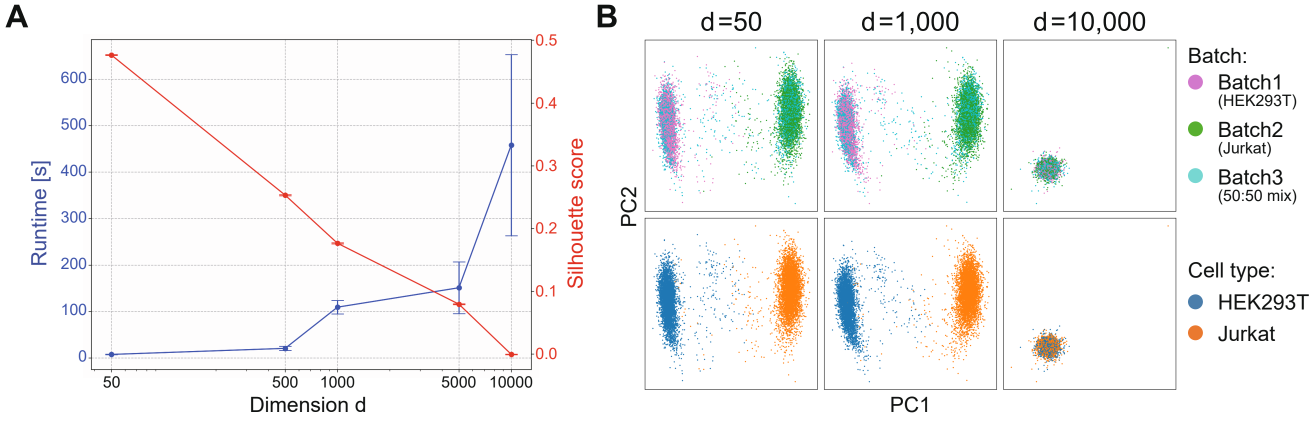

Figure S1: Impact of dimensionality on the performance of batch correction in single-cell data analysis, related to Figure 1. (A) A dual-axis plot demonstrating the performance of the batch correction method, Harmony, on scRNA-seq data from three batches containing HEK293T and Jurkat cell lines. The computational runtime, indicated by the blue line and plotted against the left vertical axis, shows an exponential increase with the dimensionality of the data. Conversely, the silhouette score for cell type, indicating the accuracy of batch correction, depicted by the red line and plotted against the right vertical axis, decreases as dimensionality increases, indicating that the batch correction method is also affected by the curse of dimensionality. The error bars represent standard deviations from 10 independent computations. (B) PCA projections illustrating the separation of cell types and batches at different data dimensionality. The plots reveal that higher dimensionality can degrade the clarity of cell type discrimination within batch-corrected data. These findings emphasize the necessity of dimensionality reduction as a preprocessing of batch correction, highlighting the difficulty in combining batch correction and technical noise reduction methods.

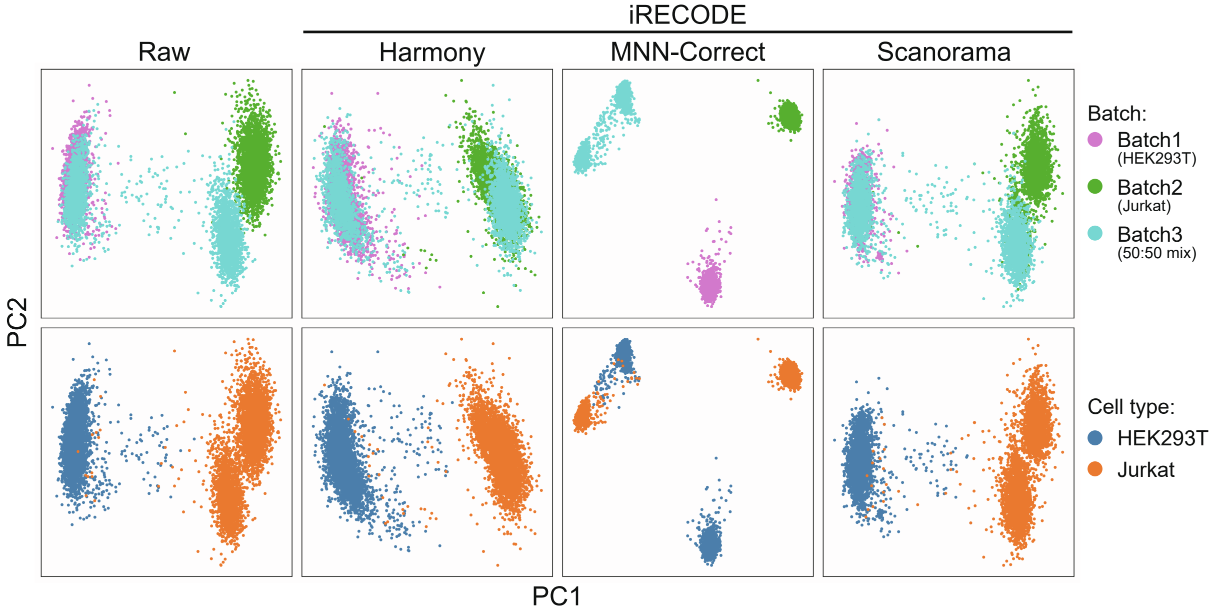

Figure S2: **Compatibility of batch correction methodologies in iRECODE, related to Figure 1.** This figure illustrates the performance of iRECODE incorporating three prominent batch correction algorithms, Harmony, MNN correct, and Scanorama, across datasets encompassing three batches and two cell lines (HEK293T and Jurkat). The first column represents the raw, uncorrected data, whereas the subsequent columns depict the data post-iRECODE using each method along the first two principal components (PC1 and PC2). iRECODE incorporating Harmony is notably effective in integrating the batches while maintaining cell type distinctions, as evidenced by the aligned clusters that segregate according to cell type rather than batch, underscoring its utility in harmonizing single-cell data while preserving biological variability.

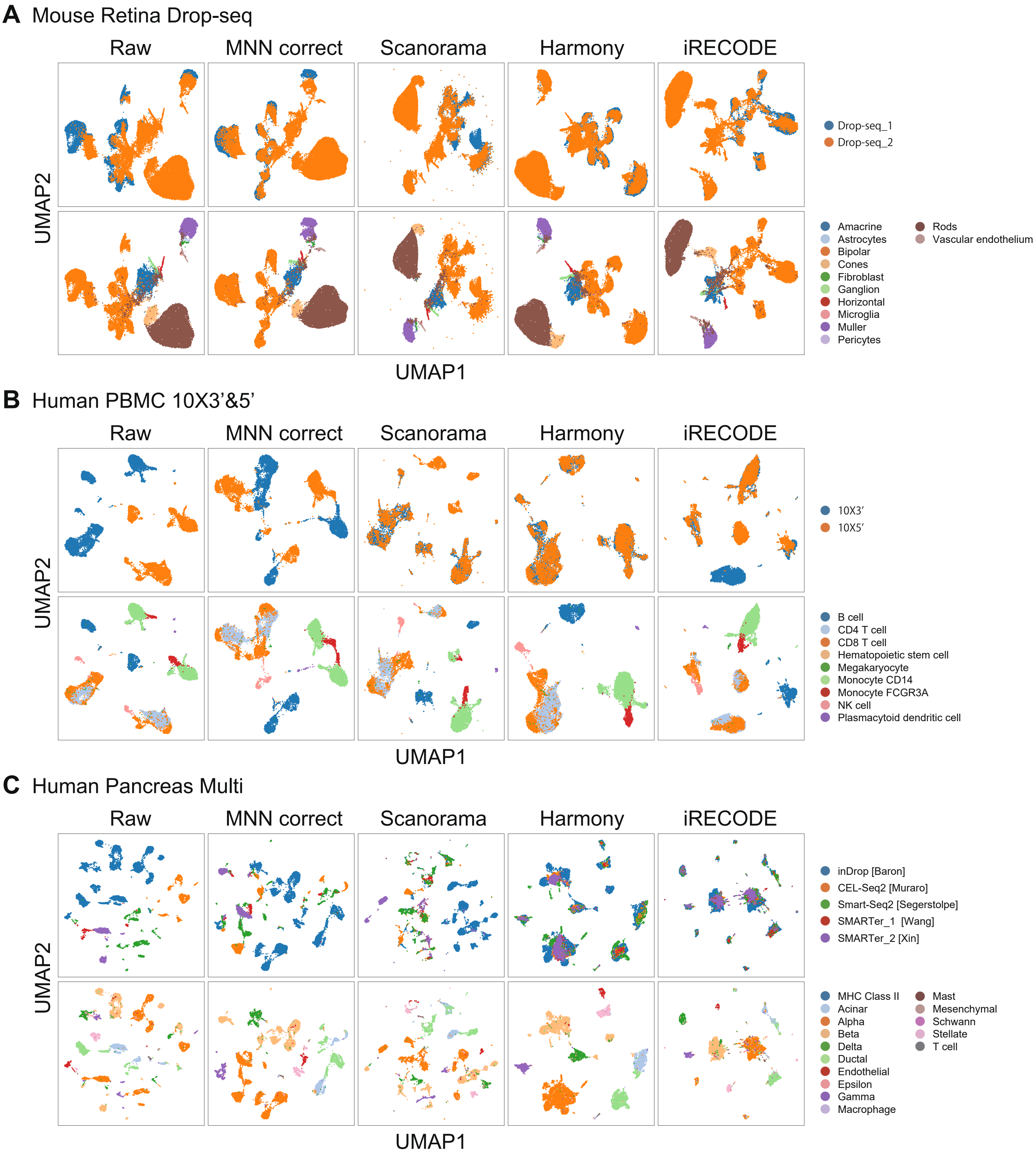

Figure S3: **Comparative visualization of scRNA-seq data integration using iRECODE across multiple datasets and platforms, related to Figure 2.** (A and B and C) UMAP projections demonstrating batch and cell-type distribution within mouse retina data obtained via Drop-seq (A) human peripheral blood mononuclear cells (PBMC) sequenced using 10X Genomics' 3' and 5' chemistry (B) and human pancreas cells sequenced using multiple single-cell technologies (C) with and without batch correction by various algorithms, including MNN correct, Scanorama, Harmony, and iRECODE.

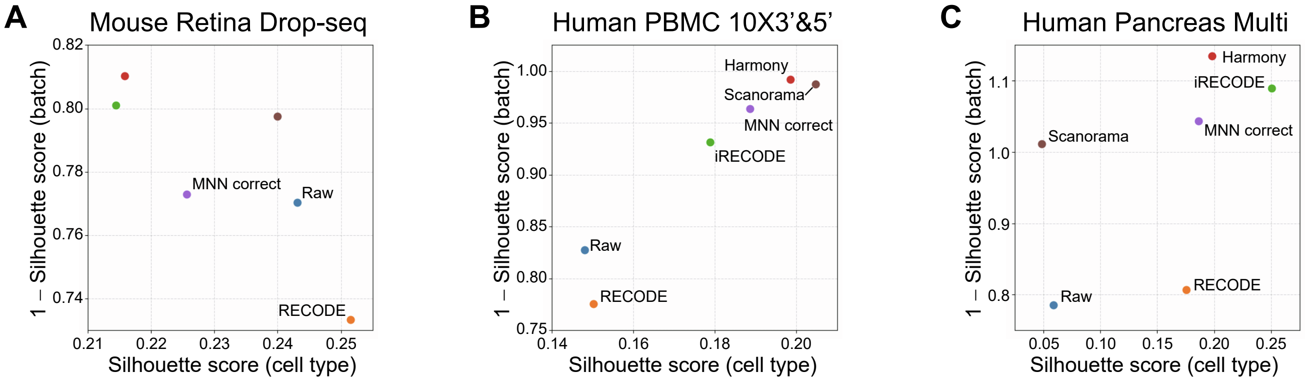

Figure S4: **Quantitative assessment of batch correction efficacy using silhouette scores across multiple datasets in Figure S3, related to Figure 2.** (A, B and C) Scatter plot illustrating the silhouette scores for cell type versus batch correction in the mouse retina Drop-seq dataset (A) the human PBMC 10X 3' and 5' dataset (B) and the human pancreas multi-platform dataset (C). Various batch correction methods, including MNN correct, Scanorama, Harmony, and iRECODE, are compared against the raw and original RECODE. Each point represents the score of a particular method, with the proximity to the top right indicating superior batch correction performance. While iRECODE consistently outperforms the original RECODE, its performance, like that of other batch correction methods, can vary depending on the dataset. This observation suggests that the effectiveness of iRECODE, as well as that of conventional batch correction approaches, depends on the specific characteristics and quality of the data, highlighting the importance of selecting appropriate methods based on the dataset context.

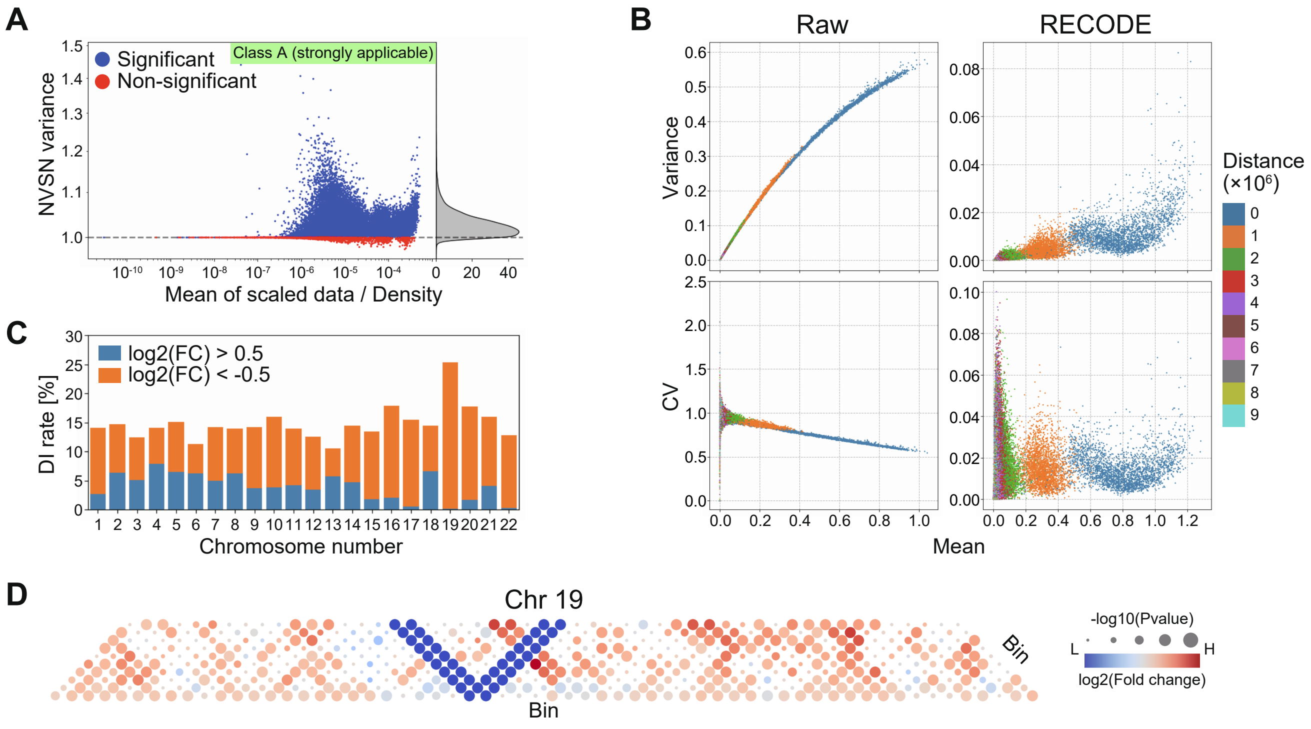

Figure S5: **scHi-C data analysis via RECODE, related to Figure 4.** (A) NVSN plot demonstrating RECODE's suitability for noise reduction of scHi-C data. (B) Comparative plots of variance and coefficient of variation (CV) against the mean for raw and RECODE-processed scHi-C data, with color gradation representing inter-bin distances. (C) Bar chart depicting the percentage of bins within differential interaction (DI) regions across all chromosomes, differentiated by the direction of log2 fold change (FC), providing insights into the chromosomal distribution of DIs defined in Figure 4F. (D) Spatial representation of DIs along chromosome 19, with circle size indicating the magnitude of $-\log(p\text{-value})$ and color denoting log2(FC), visualizing the regions of significant chromosomal interactions. These visualizations collectively quantify the impact of RECODE processing on the clarity and interpretability of scHi-C data, facilitating a deeper understanding of chromatin organization.

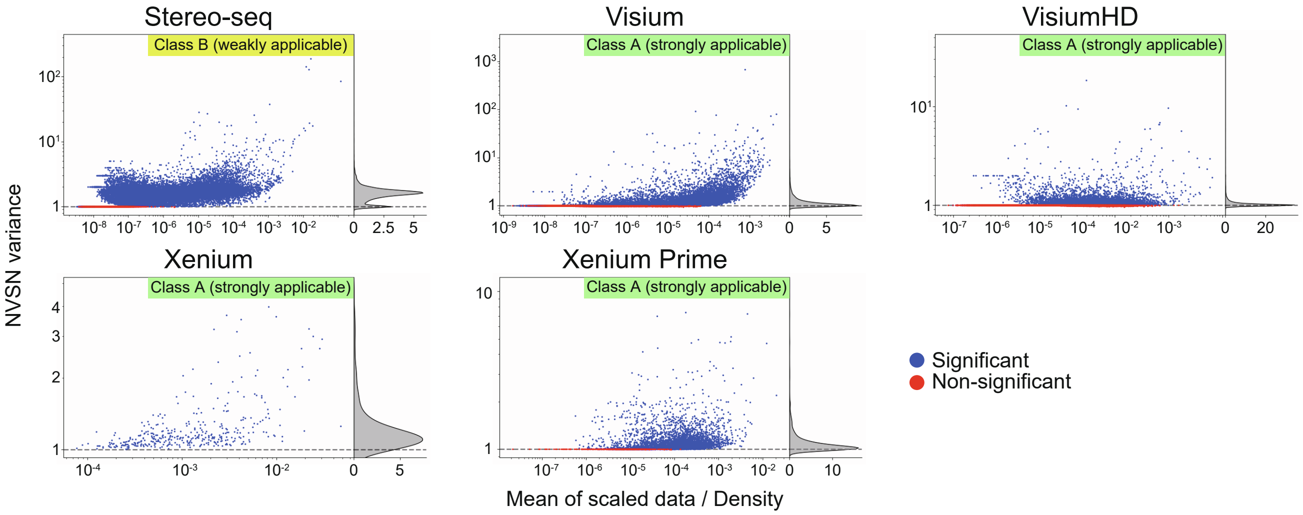

Figure S6: **Assessment of RECODE applicability to spatial transcriptomics datasets, related to Figure 5.** Spatial transcriptomics data from 10X Visium, 10X Visium HD, 10X Xenium, and Xenium Prime were classified as Class A (strongly applicable), whereas data from Stereo-seq were classified as Class B (weakly applicable).
